# Preliminary insights on the metabolomics of *Trichinella zimbabwensis* infection in Sprague Dawley rats using GCxGC-TOF-MS (untargeted approach)

**DOI:** 10.3389/fmolb.2023.1128542

**Published:** 2023-02-17

**Authors:** I. S. Ndlovu, Ekuyikeno Silas, S. I. Tshilwane, M. Chaisi, A. Vosloo, S. Mukaratirwa

**Affiliations:** ^1^ School of Life Sciences, University of KwaZulu-Natal, Westville Campus, Durban, South Africa; ^2^ Department of Veterinary Tropical Diseases, Faculty of Veterinary Science, University of Pretoria, Pretoria, South Africa; ^3^ Foundational Biodiversity Science, South African National Biodiversity Institute, Pretoria, South Africa; ^4^ One Health Center for Zoonoses and Tropical Veterinary Medicine, School of Veterinary Medicine, Ross University, Basseterre, Saint Kitts and Nevis

**Keywords:** gas chromatographic time of flight mass spectrometry (GCxGC-TOF/MS), trichinellosis, metabolomics, serum, *Trichinella zimbabwensis*

## Abstract

*Trichinella* infections have been documented globally and have been detected in wild and/or domestic animals except Antarctica. There is paucity of information in the metabolic responses of hosts during *Trichinella* infections and biomarkers for infection that can be used in the diagnosis of the disease. The current study aimed to apply a non-targeted metabolomic approach to identify *Trichinella zimbabwensis* biomarkers including metabolic response from sera of infected Sprague-Dawley rats. Fifty-four male Sprague-Dawley rats were randomly assigned into *T. zimbabwensis* infected group (*n* = 36) and the non-infected control (*n* = 18). Results from the study showed that the metabolic signature of *T. zimbabwensis* infection consists of enriched methyl histidine metabolism, disturbance of the liver urea cycle, impeded TCA cycle, and upregulation of gluconeogenesis metabolism. The observed disturbance in the metabolic pathways was attributed to the effects caused by the parasite during its migration to the muscles resulting in downregulation of amino acids intermediates in the Trichinella-infected animals, and therefore affecting energy production and degradation of biomolecules. It was concluded that *T. zimbabwensis* infection caused an upregulation of amino acids; pipecolic acid, histidine, and urea, and upregulation of glucose and meso-Erythritol. Moreover, *T. zimbabwensis* infection caused upregulation of the fatty acids, retinoic acid, and acetic acid. These findings highlight the potential of metabolomics as a novel approach for fundamental investigations of host-pathogen interactions as well as for disease progression and prognosis.

## 1 Introduction

Trichinellosis is an important zoonotic disease that is caused by a tissue-dwelling nematode that belongs to the genus *Trichinella* (Murrell and Pozio, 2011). Additionally, trichinellosis is not only a public health hazard affecting human patients but also an economic problem to food safety and mainly to pork production and trade (Gottstein et al., 2009). Cases of *Trichinella* infection are reported to be globally distributed with the exception of Antarctica (Mukaratirwa et al., 2013). Close to 11 million people globally are at risk and occur in 66 countries (Dupouy-Camet, 2000). In sub-Saharan Africa, the increase in the consumption of bushmeat, bushmeat trade, and implementation of Transfrontier Conservation Areas (TFCAs) may increase the risk of the disease in humans (Mukaratirwa et al., 2013). After the consumption of raw or undercooked infected meat, muscle larvae develop into adult worms, in the small intestines, thereafter, releasing newborn larvae that migrate to the muscles (Pozio, 2018). No clinical signs have been reported in infections in mammals except in humans and non-human primates where common signs include fever, myalgia, abdominal pain, myocarditis, and periorbital oedema (Mitreva and Jasmer, 2006; Mukaratirwa et al., 2008). After 1 week of ingestion, symptoms of infection begins where fever can persist for up to 3 weeks, depending of the severity of diseases and infection dose (Thawornkuno et al., 2022).

Ten species and three genotypes of *Trichinella* are recognized globally (Gottstein et al., 2009). The different *Trichinella* species are grouped into two major clades based on the presence or absence of a collagen capsule that surrounds the parasite while in the muscles i.e., encapsulated and non-encapsulated clades (Kocięcka, 2000). The members of the encapsulated clades, that infect only mammals, are *T. chanchalensis*, *T. nelson*, *T. nativa, T. britovi, T. patagoniensis, T. spiralis*, *T. murrelli*, and other *Trichinella* genotype T6, T8, T9. While the non-encapsulated clade includes *T. papuae*, *T. pseudospiralis* and *T. zimbabwensis* (Zarlenga et al., 2020). *Trichinella zimbabwensis* is distributed in Southern and Eastern areas of Sub-Saharan Africa and has been reported in Mozambique, South Africa, Zimbabwe, and Ethiopia. The parasite has the potential to cause future outbreaks of human trichinellosis in sub-Saharan Africa due to its capability to infect non-human primates, poor animal husbandry (especially pigs), increased animal movement and increased human-wildlife interaction (Gottstein et al., 2009; Mukaratirwa et al., 2013). In humans, the differential diagnosis must be conducted either before or during acute stages of infection so that treatment can be administered promptly.

In the past two decades, more research has been conducted on pathological effects and immunological responses of the encapsulating species, *T. spiralis*, and less work has been done on the non-encapsulating *T. zimbabwensis* (Onkoba et al., 2016). Moreover, few studies have provided a global view of changes in the metabolic serum profile of *Trichinella*-infected individuals. This remains a challenge as, during the developmental stages of the parasite within its host, numerous metabolites are exchanged and produced between the host and the parasite (Chaijaroenkul et al., 2011). The paucity of information on the metabolic response that is induced by *T. zimbabwensis* in their host has hindered efforts to advance the diagnosis, management, control, and surveillance of the parasite. Additionally, there are no studies that have applied metabolomics to identify serum biomarkers for *T. zimbabwensis* infection.

Therefore, there is a need to understand the underlying metabolic changes induced by the parasite during infection with the aid of metabolomics, which is a systematic study of metabolites in an organism (Aderemi et al., 2021). Furthermore, metabolomics has been used to diagnose various diseases such as cardiovascular diseases, and cancer, including parasite-host interaction and biomarkers discovery responses to treatments, and identification of metabolic pathways that are perturbed during infections (Halama 2014; Maia et al., 2020). Metabolic profiling approaches have been used in several helminthic parasites such as *Fasciola hepatica* and *Onchocerca volvulus* (Denery et al., 2010; Saric et al., 2010). Therefore, an investigation into the metabolic responses of rats infected with *T. zimbabwensis* is needed to identify potential diagnostics markers. Additionally, a reliable and non-invasive biomarker has not been identified or reported for the early diagnosis of *T. zimbabwensis*.

Given the above, the aim of this study was to apply a non-targeted metabolomic approach to identify *T. zimbabwensis* infection biomarkers from serum samples of infected Sprague Dawley rats using comprehensive two-dimensional gas chromatography coupled with a time-of-flight Mass-Spectrometry (GCxGC-TOF/MS). Furthermore, results from the study will contribute to elucidating the metabolic response of the host to *T. zimbabwensis* infection.

## 2 Materials and methods

### 2.1 Experimental design

**FIGURE 1:**
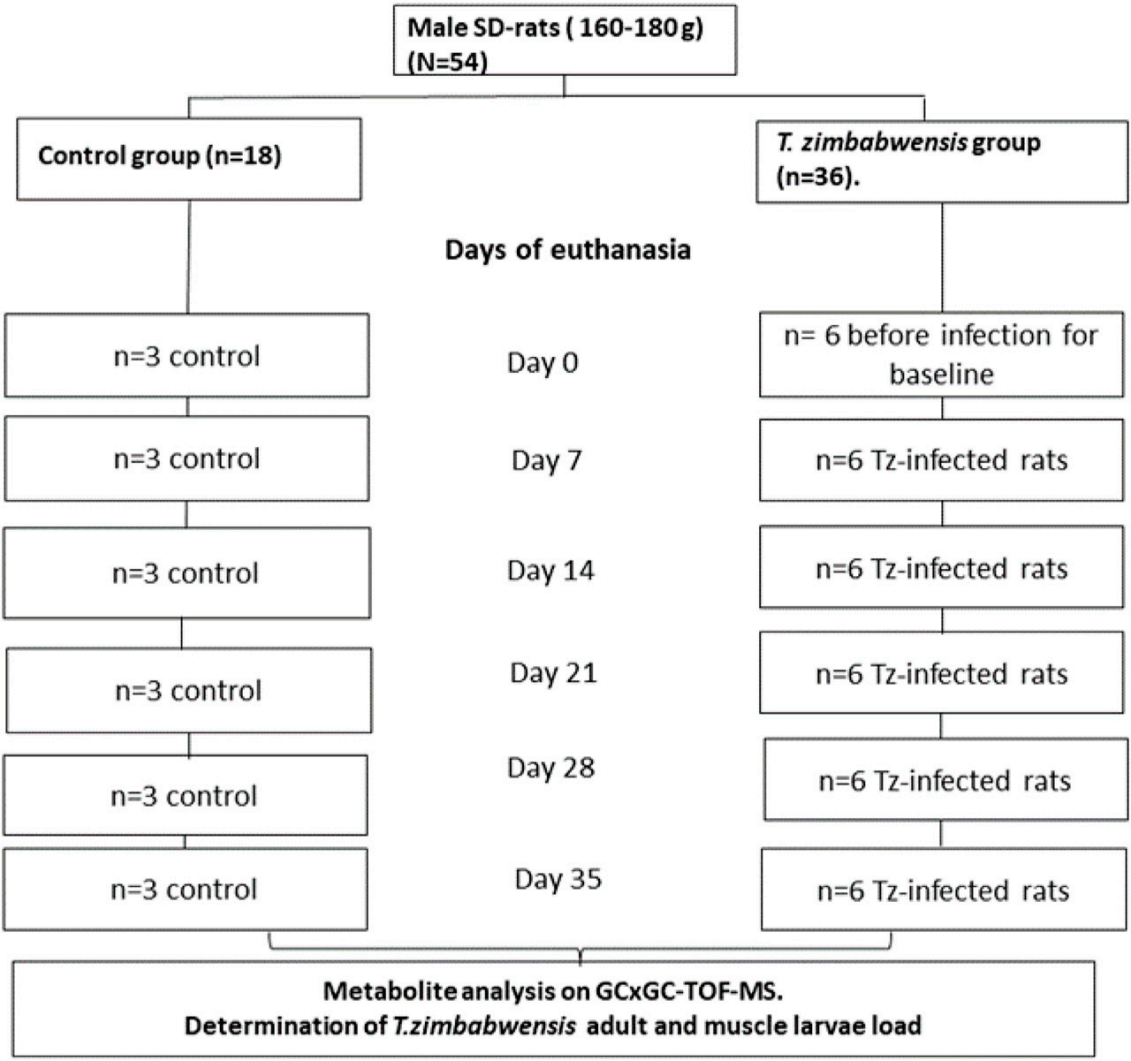
Schematic diagram of the experimental design. SD = Sprague-Dawley; *T. zimbabwensis = Trichinella zimbabwensis,* T.z -infected = *Trichinella zimbabwensis* infected.

Fifty-four male Sprague-Dawley rats weighing between 160–180 g were randomly assigned into two experimental groups, namely, *T. zimbabwensis* infected group (Tz) (*n* = 36) and the non-infected control group (*n* = 18). Rats were euthanized using isofor inhalation in a gas chamber at day 0, 7, 14, 21, 28, and 35 post-infections (pi). At each day of sacrifice, six rats were euthanized from the infected group while three rats were euthanized from the control group. On each day of sacrifice, blood samples were collected from rats *via* cardiac puncture to obtain serum. Serum samples were analyzed using an untargeted metabolomics approach with a two-dimensional Gas Chromatographic Time of Flight Mass Spectrometry (GCxGC-TOF/MS) (Figure 1). To ensure precision and accuracy of the data obtained, Quality Control (QC) samples were used to check the stability of the instrument and the reliability of the method. For the QC, a pooled QC was compiled by combining 100 μL sample from each sample group. The schematic diagram of procedures followed in the identification of compounds and pathway analysis is depicted in Supplementary Figure S1.

### 2.2 Ethical consideration

The study was conducted at the Biomedical Resource Unit (BRU), University of KwaZulu-Natal, Westville Campus, Durban, South Africa. All experimental protocols and procedures of the present study were reviewed and approved by the animals ethics committee of the University of Kwa-Zulu Natal under the reference number AREC/028/018D.

### 2.3 Infection of rats with *T. zimbabwensis* and parasite isolation

Rats were infected with crocodile-derived *T. zimbabwensis* strain (Code ISS1209) larvae obtained from whole eviscerated carcasses of infected stock rats maintained at the Biomedical Research Unit, Westville Campus, University of KwaZulu-Natal. Carcasses were digested following the protocol described by Kapel and Gamble (2000). At day 0, six animals from Tz-infected group (before they were infected) were euthanized to serve as a baseline. On day 0 the remaining 30 animals of the Tz-infection group were infected with three muscle larvae per Gram (lpg) of body weight *via* oral dosage as described by Mukaratirwa et al. (2003). Thereafter, at each day i.e., 7, 14, 21, 28, and 35 post-infection (pi), six rats were euthanized from the *T. zimbabwensis*-infected group, and intestines from the euthanized rats were separated and processed to recover adult worms (AW) as described by Mukaratirwa et al. (2003) while the whole carcass was digested for muscle larvae recovery. Three rats from the non-infected group were euthanized at each day as control.

### 2.4 Analysis of serum samples

Blood samples were collected from Tz-infected and control groups *via* cardiac puncture for serum at day 0, 7, 14, 21, 28, and 35. A total of 54 serum samples (2 mL) were collected from individual rats in the Tz-infected and control groups and then stored at −80°C in a Bio Ultra freezer (Snijders Scientific, Tilburg, Netherlands) until they were transported to North-West University, (Potchefstroom, South Africa), Center for Human Metabolomics for analysis using a GCxGC-TOF/MS.Each serum sample was considered a biological replicate. Whole metabolome was extracted from all the serum samples by applying the protein crash method. Briefly, serum samples were aliquoted in 1.5 mL Eppendorf tubes, 50 μL of the internal standard (3-phenyl butyric acid 50 ppm) was added to 50 μL of each serum sample, and 300 μL ice-cold acetonitrile was added, vortexed, and incubated on ice for 10 min. After incubation, the sample mixtures were centrifuged at 13,000 rpm for 10 min at 4°C. The supernatant was transferred into a GC vial and dried under a gentle stream of nitrogen at 40°C for 20–30 min. For derivatization purposes, 50 μL methoxyamine HCl (150 mg in 10 mL pyridine) was added followed by an incubation step at 50°C for 90 min. Thereafter, 40 μL BSTFA +1% TMCS was added, and the extract was re-incubated at 50°C for 60 min. The 50 μL extracts were then transferred to a 250 μL insert in a sample vial and capped before GCxGC-TOF-MS analysis.

### 2.5 Untargeted GCxGC-TOFMS approach

#### 2.5.1 GCxGC-TOFMS analysis

Pegasus GCxGC-TOFMS (Leco Corporation Joseph, MI, USA) that uses an Agilent 7890A GC (Atlanta, GA) coupled to a time-of-flight mass spectrometer (TOFMS) (Leco Corporation, St Joseph, MI, USA) equipped with a Gerstel Multipurpose sampler, was used for chromatographic analyses of the derivatized samples. One µL of serum extract was randomly injected at a split ratio of 1:50 and the carrier gas used was helium at a flow rate of 1 ml min^−1^. For the entire run, the temperature of the injectors was kept constant at 270°C. A Restek Rxi-5Sil MS capillary column (29.145 m × 0.25 μm d.f.) was used as the primary column. The primary oven was programmed to an initial temperature of 70°C for 2 min to obtain a compound separation. Subsequently, this was followed by a 4°C per minute increase to a final temperature of 300°C where it was maintained for 2 min. The second separation of compounds was achieved using a Restek Rxi-17 (1.400 m, 0.25 µm i.d., 0.25 μm d.f.) column. The secondary column used was set to the same temperature parameters as that of the primary column. The filament bias was EI at 70 eV while the detector voltage was at 1,600 V. Subsequently, the mass spectra were collected at an acquisition rate of 200 spectra per second with a source temperature of 220°C and a solvent delay of 400 s from 50 to 800 m/z (Du Preez and Loots 2013).

#### 2.5.2 Peak identification

Leco Corporation Chroma-TOF software (version 4.50) was used to obtain peak finding and mass spectral deconvolution at an S/N ratio of 100, with a minimum of three apexing peaks. Using the mass fragmentation patterns generated by the MS, together with their respective GC retention times, the identities of these peaks were determined by comparing them to commercially available NIST spectral libraries (mainlib, replib).

#### 2.5.3 Data clean-up

Data clean-up was conducted using Microsoft Excel to remove features that were not reliably measured. The reliability of each variable was assessed by calculating the relative standard deviation (standard deviation divided by the mean) across all quality control samples of Tz and control samples. Features with a relative standard deviation above 50% were excluded from further analysis. This was accomplished by replacing all zero values with half the minimum observed value for the dataset, which served as an estimate for the detection limit. The data were log-transformed and scaled using MetaboAnalyst where data were not normally distributed. Natural shifted log transformation was performed to correct the skewness distribution of the variables, followed by auto-scaling to place all variables to obtain normality (Du Preez and Loots 2013).

### 2.6 Identification of compounds and pathway analysis

The potential biomarkers were selected based on the greatest variable importance in the projection (VIP) value and had to be statistically significant (VIP >1.5 and *p* < 0.05) (Alonso et al., 2015; Sun et al., 2017). The identified metabolic biomarkers were mapped onto the Kyoto Encyclopedia of Genes and Genome (KEGG) pathway network using iPath 3.0 (http://pathways.embl.de), a web-based application used to visualize and analyze cellular pathways. A metabolite set enrichment overview (MSEA) was constructed using the significant metabolites to elucidate the pathways that these significant metabolites are involved in using MetaboAnalyst version 5.0 (https://www.metaboanalyst.ca/docs/Publications.xhtml), an online tool for analyzing and interpreting metabolic enrichment. Moreover, Metabolic Pathway Analysis (MetPA) was incorporated with MetaboAnalyst for pathway analyses.

### 2.7 Statistical analysis

Data were analyzed by using MetaboAnalyst version 5.0 and the graphical representation of data was on Prism software Version 5. The integral intensity of the metabolites was presented as median with Inter Quartile Range (IQR). Prior to Principal Component Analysis (PCA), data were normalized using auto-scaling and log transformation. For data overview and pattern discovery, a PCA was first constructed followed by a supervised classification method, Partial Least-Squares Discriminant Analysis (PLS-DA). Thereafter, a supervised orthogonal partial least squares discriminant analysis (OPLS-DA) method was performed to improve the separation between the group of samples and to minimize other biological analytical variations. The OPLS-DA was cross-validated by permutation (*n* = 20). Thereafter, the goodness-of-fit parameters were then calculated (R2X, R2Y and Q2Y). The receiver-operating curve analysis (ROC) was used to further evaluate the predictive ability of the identified potential metabolic biomarkers. Moreover, the Area under the curve was used to determine the diagnostic accuracy of the biomarkers where 0.8 < AUC <0.9 was considered good and 0.9 < AUC ≤1.0 considered excellent. To assess if there was a significant difference (*p* < 0.05) in the levels of potential biomarker metabolites at different days, one-way analysis of variance (ANOVA) was performed followed by *post hoc* analyses using Fisher’s LSD. For quality assurance, aliquots from the pooled QC samples were used to determine if the samples were consistently analyzed within and across batches. A PCA score plot was used to ensure the absence of possible batch effects in the generated data.

## 3 Results

### 3.1 Parasite establishment

**FIGURE 2:**
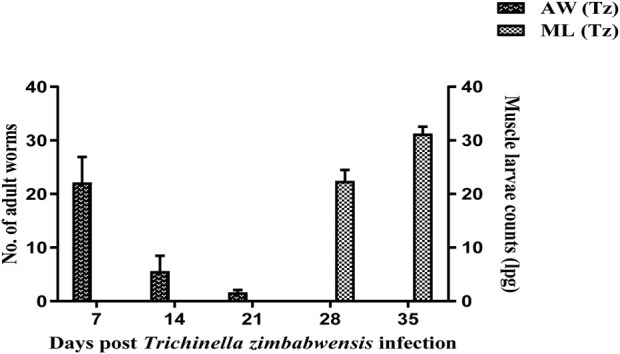
Mean number (± SD) of intestinal adult worms and muscle larval counts recovered from Sprague Dawley rats infected with *T. zimbabwensis.* AW = Adult worms; ML = Muscle larvae.

In the intestine of the Tz-infected rats, adult worms were recovered at day 7, 14, and 21 pi (Figure 2). Moreover, there was a high number of recovered adult worms at day 7 compared to day 14 and 21 pi. Larvae in muscle were first detected at day 28 pi and at day 35 pi at a rate of 23 and 31 lpg of muscle respectively.

### 3.2 Quality assurance

Quality assurance was carried out using a PCA, to determine any variation between the QC and that of the serum samples analyzed. Supplementary Figure S2 in the supplementary material depicts the score plot of the three PCs, with PC1 explaining 18%, PC2 explaining 12.6%, and PC3 explaining 7.4%.

### 3.3 Discrimination between *T. zimbabwensis*-infected rats and control rats

**FIGURE 3:**
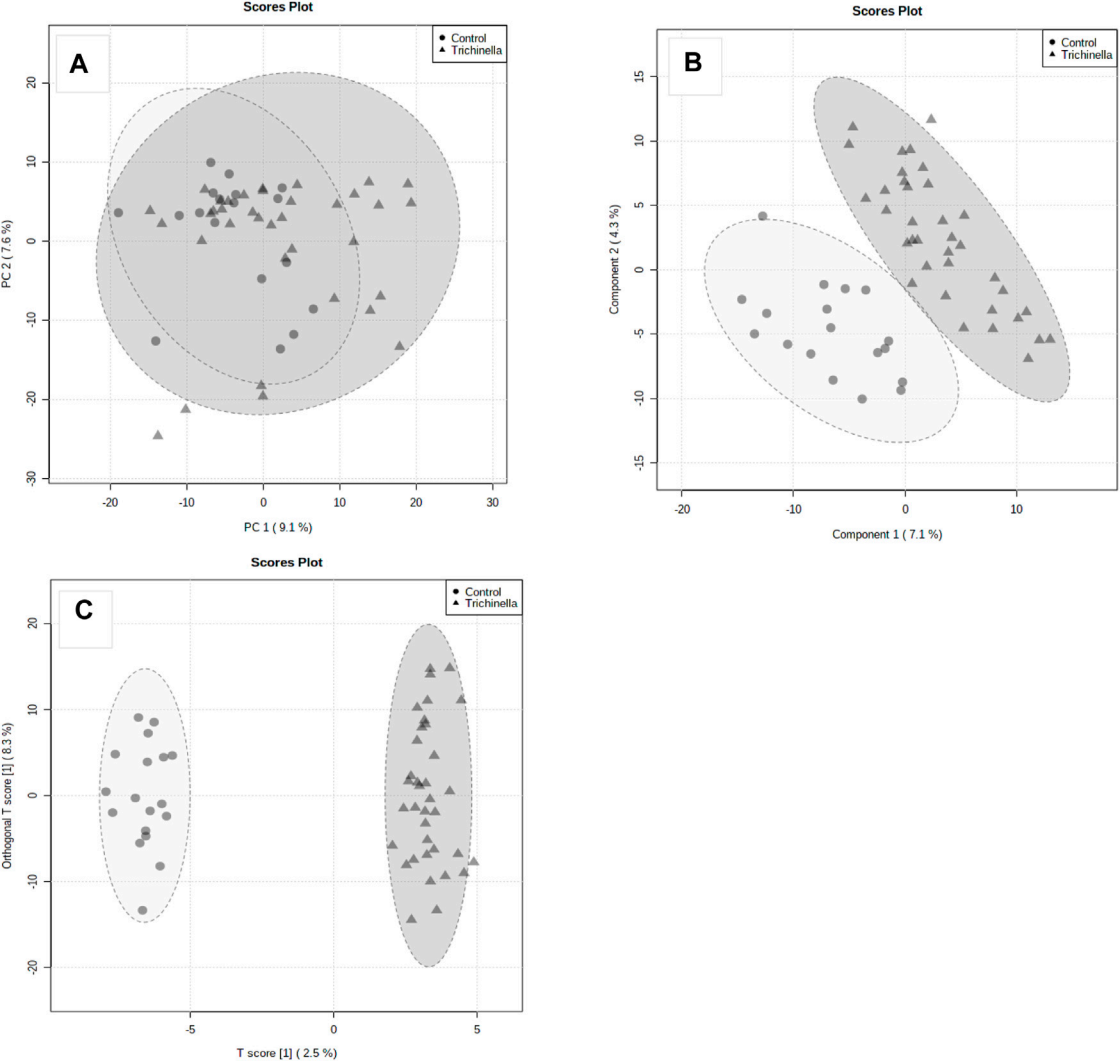
Principal Component Analysis (PCA) **(A)**, PLS Discriminant Analysis (PLS-DA) **(B)**, and **(C)** Orthogonal PLS-DA score plots. The score plots, the abscissa PC1, and the ordinate PC2 represent the scores of the principal components ranking the first and the second, respectively, and different shapes of the scattered points represent the different groups of the samples. The explained variances are shown in brackets. The Orthogonal PLS-DA score plot **(C)** of Tz-infected rats (Grey triangles) and the control rats (Grey circles).

A total of 941 metabolites were identified in the serum samples from the experimental animals. Using fold change analysis, from the 941 identified metabolites, 28 metabolites were significantly upregulated, 87 metabolites were downregulated and 829 were not significantly changed. Considering the unsupervised PCA results using all 941 metabolites and all serum samples, there was moderate natural discrimination between the Tz-infected and the control group (Figure 3A). The interpretation rate of the first principal component (PC1) and the second principal component (PC2) were 9.1% and 7.6%, respectively. The difference was observed between the two groups on the PLS-DA score plot (Figure 3B). The distinction between the two groups was further evaluated using the orthogonal projections to latent structures–discriminant analysis (OPLS-DA) plot, which was constructed using the 941 metabolites (Figure 3C). The OPLS-DA (*R* ^2^ = 0.85; Q^2^ = 0.07) depicted a clear distinction between the Tz-infected and the control group (Figure 3C). Additionally, the good discrimination between the Tz-infected and the control group indicated that the two groups had distinct metabolic profiles. The goodness-of-fit of the parameters for the OPLS-DA, R2X, R2Y, and Q2Y were then calculated. R2X and R2Y represented the fraction of variance of X and Y variables of the model while Q2Y represents the predictive performance of the model. The values of R2Y and Q2Y for the model were 0.98 and 0.80, respectively. All the samples were within the 95% confidence interval. The separation of the two groups showed that *T. zimbabwensis* infection caused notable metabolic changes in the infected animals.

The metabolites that had a significant change (*p* < 0.05) were identified based on the absolute cut-off value of correlation coefficient and Variable Importance in Projection (VIP>1.5) and these were considered potential biomarkers. A total of 94 metabolites were determined as metabolites most contributing to the segregation between the Tz-infected group and the control group (Supplementary Table S1). Considering that VIP scores >1.5 are interpreted as being highly influential, the top 15 metabolites were considered to have an important role in distinguishing the control rats from Tz-infected rats (Table 1). Table 1 illustrates the differences in the concentrations of all the potential biomarkers between the two groups. The significant metabolites were carbohydrates (glucose, D-mannitol, and meso-erythritol), amino acids (Pipecolic acid, histidine, Amino-butyric acids, thiazole, urea, and L-tyrosine), fatty acids (acetic acid and retinoic acid) xenobiotics (silane, Furo-2,3-Pyridine) pyrimidine nucleoside (uridine) and organic acid (succinic acid) (Table 1). Glucose, meso-erythritol, and D-mannitol were up-regulated in the Tz-infected rats by 37.224, 2.358, and 2.827-folds, respectively, as compared with the control rats (Table 1). Out of the six amino acid potential biomarkers discovered, L-tyrosine was the only metabolite that was low (Table 1) and downregulated by 0.822-folds in the Tz-infected rats as compared to the control rats (Table 1). Acetic acid and L-tyrosine are two metabolites out of the 15 identified metabolites that were found to have lower concentrations in the Tz-infected rats when compared to the control rats.

**TABLE 1:** Quantitative comparison of the potential biomarkers identified from the serum samples of Sprague Dawley rats infected with *Trichinella zimbabwensis* and the uninfected control group.

Metabolite	Chemical class	Variation trend	Fold change	VIP	[C]	[TZ]	HMDB ID
Glucose	Carbohydrate	↑	37.224	3.336	L	H	HMDB0000122
Silane	Xenobiotic	↑	3.699	3.108	L	H	None
Uridine	Pyrimidine nucleoside	↑	0.273	2.976	L	H	HMDB0000296
Pipecolic acid	Amino acid	↑	0.293	2.930	L	H	HMDB0000070
Acetic acid	Fatty acid	↓	3.138	2.882	H	L	HMDB0000042
D-mannitol	Carbohydrate	↑	2.827	2.757	H	L	HMDB0000765
Furo-2,3-Pyridine	Xenobiotic	↑	2.556	2.570	H	L	None
Histidine	Amino acid	↑	0.457	2.503	H	L	HMDB0000177
Retinoic acid	Fatty acid	↑	0.471	2.367	H	L	HMDB0001852
Amino-butyric acid	Amino acid	↑	0.892	2.328	H	L	HMDB0000112
Thiazole	Amino acid	↑	3.871	2.324	H	L	HMDB0029713
Succinic acid	Organic acid	↑	2.821	2.311	H	L	HMDB0000254
L-Tyrosine	Amino acid	↓	0.822	2.304	H	L	HMDB0000158
Urea	Amino acid	↑	8.810	2.238	L	H	HMDB0000294
meso-Erythritol	Carbohydrate	↑	2.358	2.231	L	L	HMDB0094689

Key: The arrows (↑/↓) were used to show the metabolite fold change trend upregulated/downregulated compared with uninfected controls. None = HMDB and KEGG information not available. [C] [TZ]- Used to show concentration of metabolites in both groups, C- control, TZ- *Trichinella*-infected. L-low, H-high; metabolite concentration.

**FIGURE 4:**
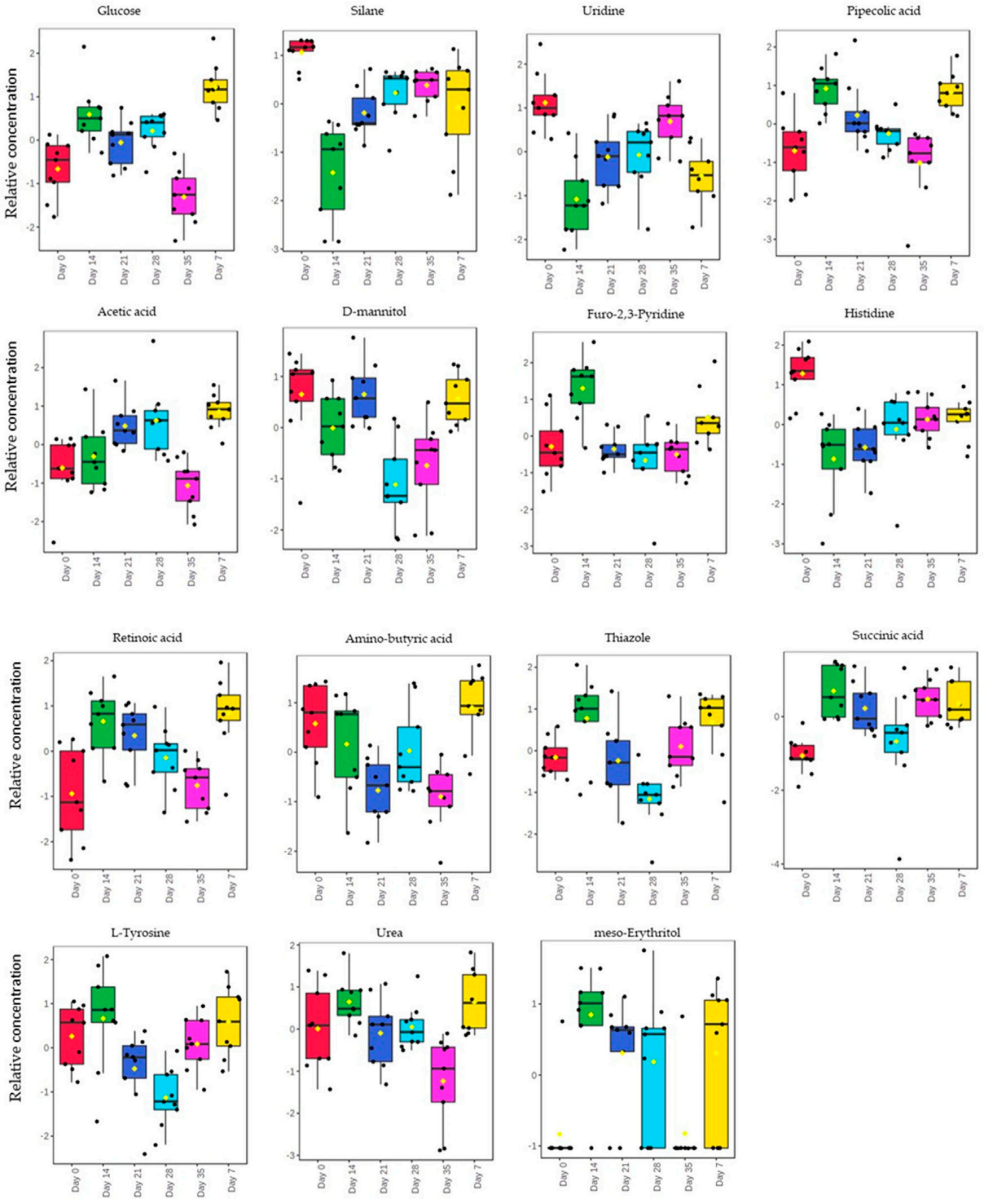
Box-Whisker plot for the significantly different metabolites (*p*-value ≤0.05). The top 15 significantly different metabolites identified and their relative concentrations of each plotted against the different days post infection.

The relative concentration of the identified biomarkers was observed to be different at different infection stages or days post-infection (Figure 4). The relative concentration of thiazole, succinic acid, uridine, d-mannitol, histidine, and L-tyrosine depicted an increase with the increase in muscle larvae, from day 28 to day 35. There was a significant difference (*p* < 0.05) in the relative concentration of glucose, pipecolic acid, furo-2,3-pyridine, amino-butyric acid, and urea between day 7, day 14 and day 21. Furthermore, these metabolites decreased from day 7 to day 21 with a decrease in the adult worms.

### 3.4 Pathway discovery and analyses

**FIGURE 5:**
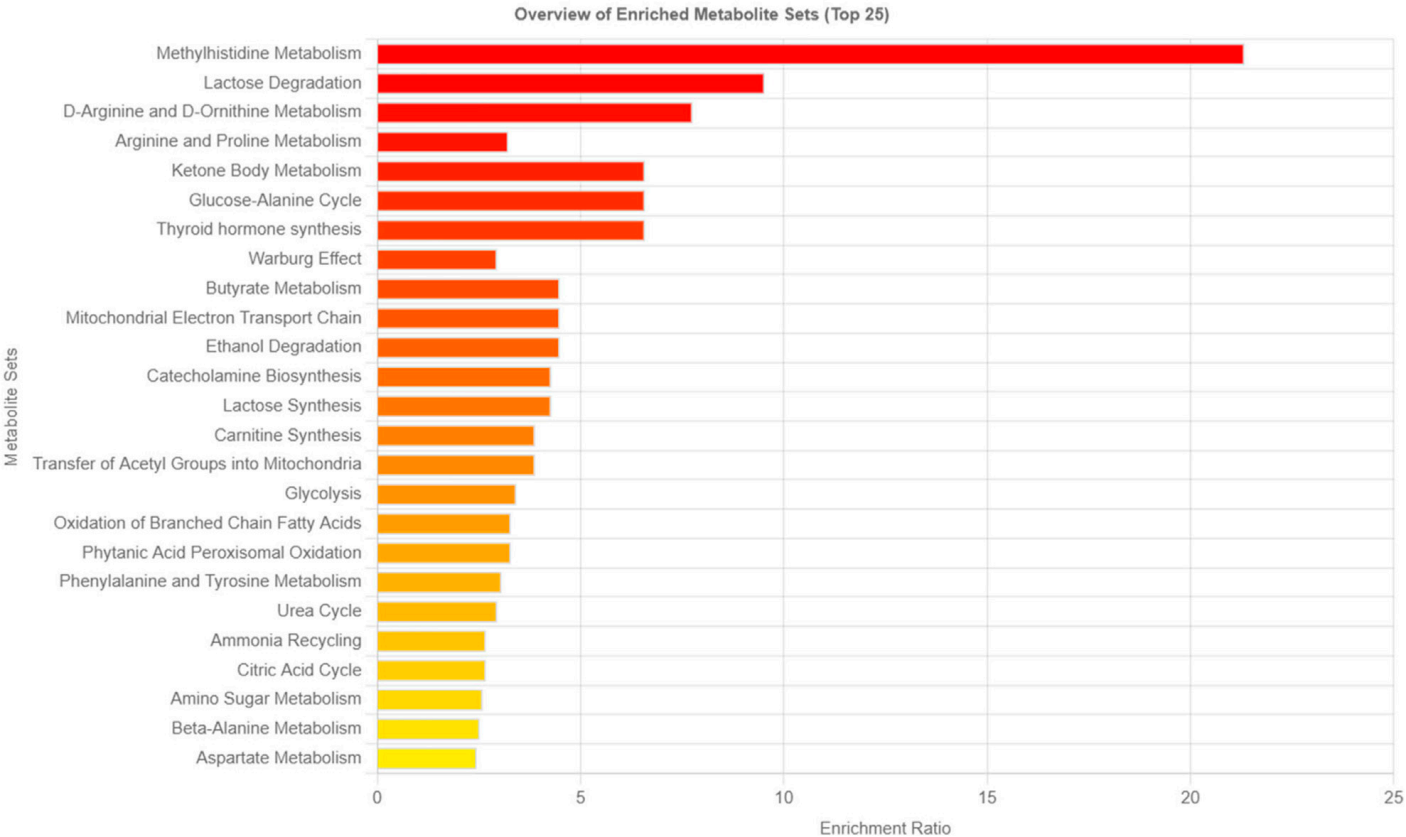
Metabolic pathways associated with the top 15 identified potential metabo-lites. The horizontal bars show a summary of metabolic pathways that were strongly affected in the *Trichinella zimbabwensis* infected group compared to the control. Color intensity (white to red) reflects increasing statistical significance.

The metabolites with log2 (FC) > 2 (fold-change) were considered significant. By conducting fold change or PLS-DA analysis, the potential to identify subtle, but consistent changes between a group of related compounds, could be undermined. Therefore, to overcome this obstacle, Metabolite Set Enrichment Analysis (MSEA) was used. MSEA plays an essential role in identifying biologically meaningful patterns that are significantly enriched in quantitative metabolomic data. Metabolic data from Tz-infected and control rats based on the top 15 biomarkers showed metabolic pathways that were significantly enriched (FDR< 0.05, p-<0.05) and these were methylhistidine metabolism, lactose degradation, D-arginine, and D-ornithine metabolism, and arginine and proline metabolism (Figure 5). Moreover, for all the 94 identified metabolites, the MSEA detected more metabolic pathways that were significantly enriched during trichinellosis infection: including urea cycle, glucose-alanine cycle, valine, leucine and isoleucine degradation amino sugar metabolism, and methylhistidine metabolism being highly enriched pathway (Supplementary Figure S3A).

**FIGURE 6:**
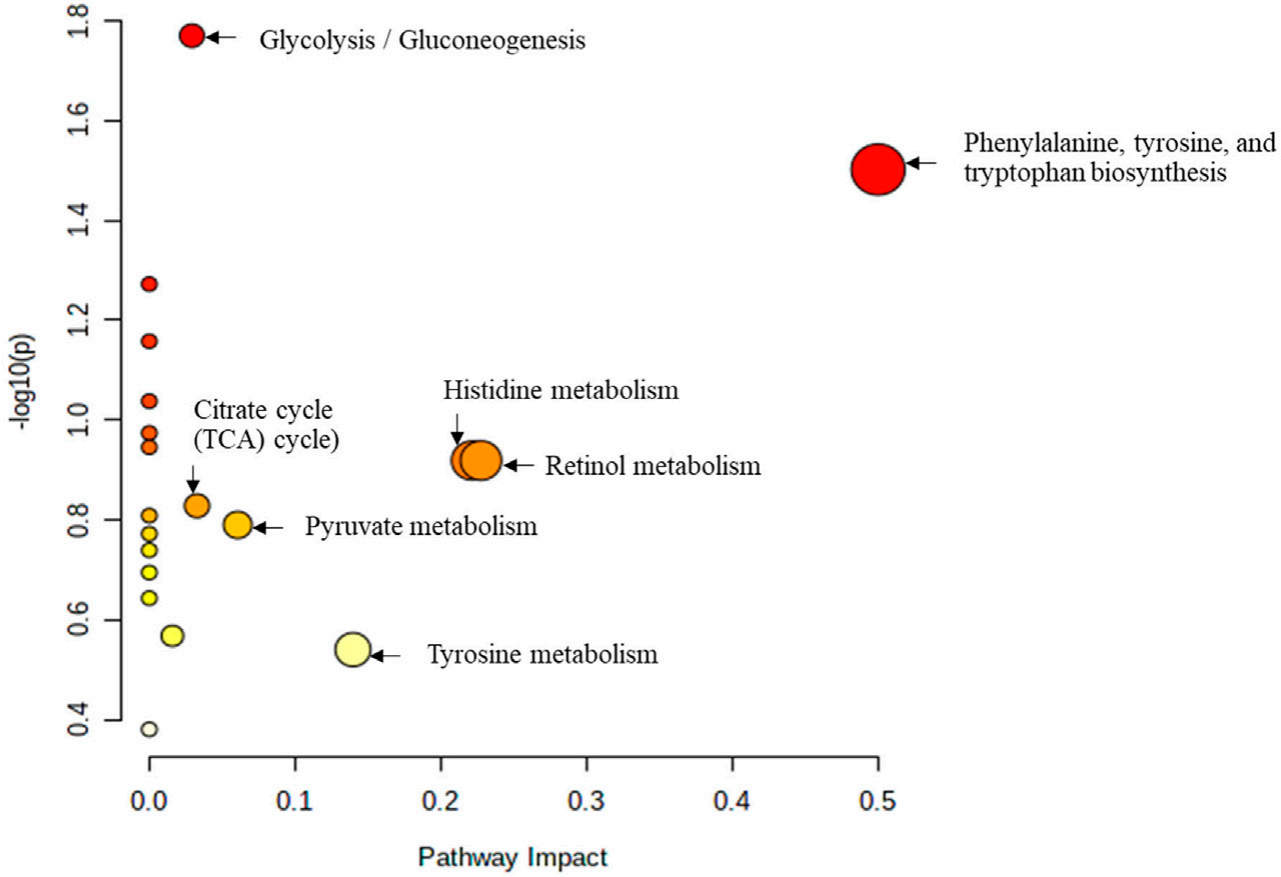
Metabolic pathways associated with the top 15 identified metabolites mark-ers. All the matched pathways are displayed as circles. The node size is proportional to the enrichment ratio. Light yellow to red indicates the *p*-value from small to large. The color and size of each circle are based on the *p*-value and pathway impact value, re-spectively. The most impacted pathways having high statistical significance scores are indicated with their names.

The study further used the Pathway analysis (MetPA), which is a module of MetaboAnalyst that combines the results from the MSEA with the pathway pathology analysis to identify the most relevant pathways in the Tz-infected group. The pathway impact analysis (*p* < 0.05 and impact values >0.1), is depicted in Figure 6 and Supplementary Figure S3B. Based on the top 15 potential markers, the most significant metabolic pathways in decreasing order were phenylalanine, tyrosine, and tryptophan biosynthesis metabolism, citrate cycle (TCA cycle), retinol metabolism, histidine metabolism, tyrosine metabolism, glycolysis/gluconeogenesis, pyruvate metabolism, and pyrimidine metabolism (Figure 6). Moreover, metabolites that were involved in these pathways are shown in Table 2 with their impact values.

**TABLE 2:** Metabolic pathways most affected during *Trichinella zimbabwensis* infection in Sprague Dawley rats from the pathway-impact analysis.

Pathway name	Compound hits	Metabolite	*p*-value	Impact
Glycolysis/Gluconeogenesis	2/26	Glucose, acetic acid	0.017*	0.029
Phenylalanine, tyrosine, and tryptophan biosynthesis	1/4	L-tyrosine	0.031*	0.500
Histidine metabolism	1/14	L-Histidine	0.120	0.221
Retinol metabolism	1/6	Retinoic acid	0.120	0.227
Citrate cycle (TCA cycle)	1/20	Succinic acid	0.042*	0.327
Pyruvate metabolism	1/22	Acetic acid	0.162	0.060
Pyrimidine metabolism	1/39	Urea	0.270	0.015
Tyrosine metabolism	1/42	L-Tyrosine	0.044*	0.139

*= Most significant metabolic pathway. Compound hits represent the number of potential biomarkers involved in each all metabolites in a pathway.

A graphical representation of the pathways identified and their relative impact for all biomarkers identified is shown in Supplementary Figure S3B. The most important pathways were arginine biosynthesis, histidine metabolism, alanine, aspartate, and glutamate metabolism and arginine and proline metabolism and these were highly impacted during *Trichinella* infection. Metabolites that were involved in each of the identified pathways are shown in Supplementary Table S3.

The identified metabolic pathways were notably associated with the establishment, development, and migration of *T. zimbabwensis* infection in the host. Moreover, differential metabolites between Tz-infected and control rats were noted in multiple pathways including energy production and supply, and maintenance of essential physiological processes, and these are mapped in the Supplementary Figure S4.

### 3.5 Verification of potential biomarkers

**FIGURE 7:**
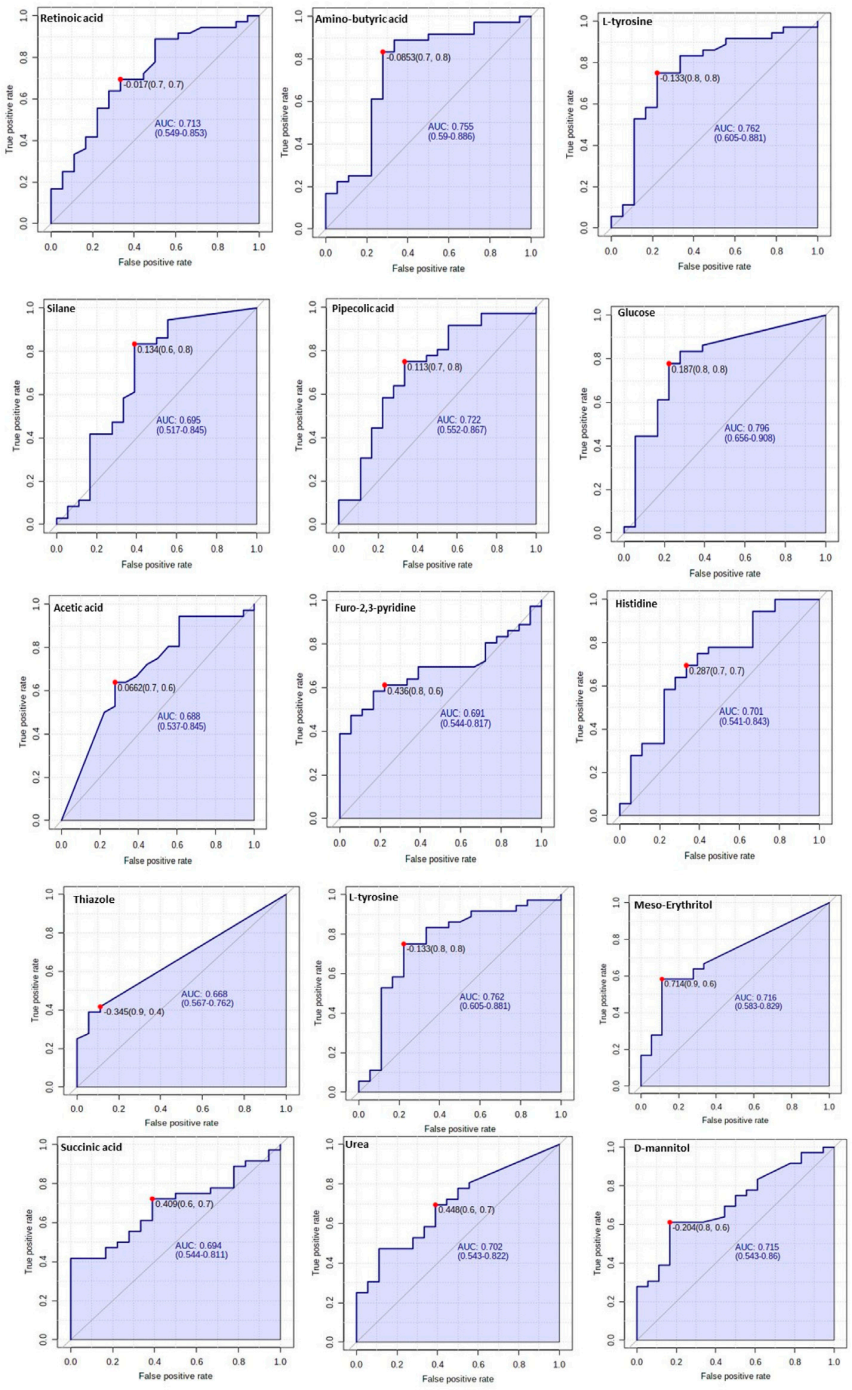
Verification of the top 15 identified potential biomarkers. ROC analysis to further depict the predictive value of these individual metabolites independently.

**FIGURE 8:**
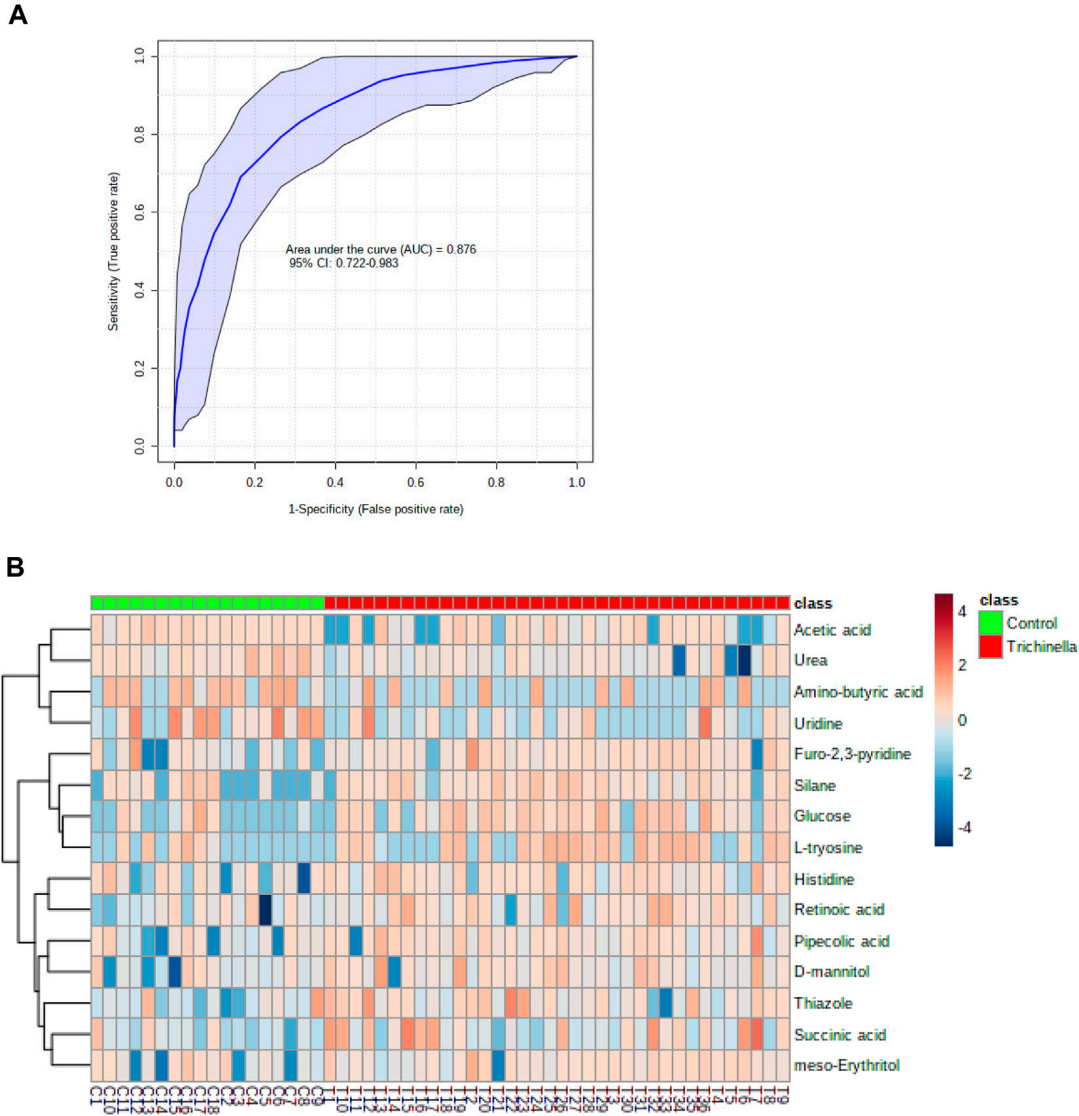
A systemic analysis of the key differential biomarkers. **(A)** The receiver-operating curve of the top 15 identified potential biomarkers for distinguishing individuals with *Trichinella zimbabwensis* (Tz) infection. **(B)** Heat map visualization of the key potential biomarkers between the Tz infected and control group.

The diagnostic performance of each of the 15 biomarkers in discriminating between the Tz-infected group and control group was evaluated using a ROC curve. Figure 7 shows the diagnostic accuracy of the identified potential biomarkers for Tz-infection. The ROC analysis showed that glucose had a high diagnostic ability with the area under the curve (AUC) value of 79.6 (*p* < 0.05) and confidence interval of 66.0–90.8, while the other metabolic makers showed an acceptable diagnostic ability. Combination of individual markers in a multivariate model showed the AUC obtained for the final model was 87.6 (95%CI 0.722–0.983) (Figure 8A), indicating a strong discriminative power and supporting the value of the identified metabolites in screening for Tz-infection. The AUC suggests that the 15 metabolites might potentially serve as biomarkers of Tz-infection. The plotted heat map showed that most metabolites were changed in the Tz-infected compared to the control with potential biomarkers showing degrees of alteration (Figure 8B). Moreover, Figure 8B showed that the Tz-infected serum samples had higher metabolite concentrations compared to the non-infected serum samples except amino-butyric acid or uridine with lower concentrations. Additionally, there were three clusters observed in the heat map with thiazole, succinic acid, and histidine, meso-erythritol forming one cluster and these in Figure 5 showed association with increase in days post-infection and increase in the muscle larval count from day 28 to day 35. Pipecolic acid on the other hand was high at the beginning of infection and then was successively decreasing till day 35.

## 4 Discussion

In this study, metabolic signature, and changes in metabolism in the Tz-infected group were observed, which showed substantial changes in the metabolome. From the top 15 potential biomarkers, six metabolites were identified as amino acids (L-tyrosine, histidine, pipecolic acid, amino-butyric acid, thiazole, and urea) and five of these except tyrosine were up-regulated in the *T. zimbabwensis* infected animals as compared to the control animals. From the MSEA, the methyl-histidine metabolism pathway was highly enriched, and the metabolite involved was histidine. Histidine is an important amino acid for most cells (Krishnan and Soldati-Favre, 2021) although, in mammals, it is not *de novo* synthesized, therefore it must be obtained through diet (Moro et al., 2020). It plays an essential role in metabolism, which includes metal-ion chelation, proton buffering, and anti-oxidative potential. In the present study, histidine concentration was higher in the *T. zimbabwensis*-infected animals as compared to the control animals. A study conducted by Holeček and Vodeničarovová (2020) on the effect of histidine supplementation in rats reported that histidine affected the concentration of other amino acids. More specifically, animals given higher histidine had high concentrations of glutamine and alanine in the muscles when compared with the control group. Glutamine is produced by the enzyme glutamine synthetase, which is involved in various biological processes and plays a role in assisting parasites to adapt to a different environment in their life cycle and make use of their host resources(Crispim et al., 2018; Holeček and Vodeničarovová, 2020). (Sun et al., 2017) found that silencing glutamine synthetase, decreased the burden of *T. spiralis* intestinal infectious larvae and impeded larval molting and development. Methylhistamine has been found in higher concentrations in the blood following skeletal muscle protein breakdown, which may result because of injury or disease (Wang et al., 2012). In the present study, this can be associated with trichinellosis.

Phenylalanine, tyrosine, and tryptophan biosynthesis were pathways which were extensively impacted by Tz-infection compared to all other metabolic pathways. Phenylalanine, tryptophan, and tyrosine are three aromatic amino acids that are involved in protein synthesis (Parthasarathy et al., 2018). In this study, tyrosine was one of the top biomarkers identified to discriminate the infected animals from the control group. In the present study, tyrosine was detected from the serum samples and was downregulated in the infected group. Looking at the metabolic differences in different infection stages, tyrosine was found to decrease from day 0 until day 21 when the adult worms were decreasing. Additionally, tyrosine increased from day 28 to day 35 post infection which is phase when the muscle larvae were increasing. The conversion of phenylalanine occurs exclusively in the liver (Møller et al., 2000). A study conducted by McAllan et al. (2013) reported that injuries in the liver can lead to changes in the amino acid metabolism, mainly manifested as an increase in the free amino acids such as tryptophan, phenylalanine and tyrosine, and a decrease in the free branched-chain amino acids. Mukaratirwa et al. (2008) reported congested liver as one of the findings in baboons and monkeys experimentally infected with *T. zimbabwensis*, and Neghina et al. (2011) have also emphasized that when considering *Trichinella* infection in humans, hepatic disturbances should be considered as liver function and protein metabolism are usually compromised. It can be speculated that the down regulation of tyrosine was due to trichinellosis as the concentration of the metabolite was higher in the control compared to the infected animals.

Other amino acids that were detected as biomarkers include L-ornithine, L-glutamate, L-glutamine, urea, L-leucine, and hydroxyproline. L-ornithine and urea are products of metabolisms of L-arginase where the enzyme arginase plays a crucial role (Durante et al., 2007). Moreover, arginase is also important in the urea cycle in the liver (Hanedan et al., 2015). In the present study, urea was up-regulated while L-ornithine was down regulated in the *T. zimbabwensis*-infected animals. Additionally, urea was involved in three metabolic pathways including D-arginine and D-arginase metabolism, urea cycle, as well as arginine and proline metabolism. Trichinellosis negatively impacted the urea cycle due to the downregulation of L-ornithine which is supposed to be transported back into the mitochondria *via* ornithine-citrulline transporter where the cycle can start again (Stone et al., 2022). The liver urea cycle plays an important role in removing toxins or detoxifying the amino groups i.e., ammonia (Zhao et al., 2022). A reaction between carbamoyl phosphate and L-ornithine forms citrulline, which plays a role in channeling ammonia into the urea cycle. According to Wijdicks (2016), insufficient removal of ammonia results in hyperammonemia and toxicity of the central neuron system, leading to hepatic encephalopathy. The study by Dupouy-Camet and Bruschi (2007) reported that *Trichinella* migration to the muscles results in the disturbance of metabolites in the host, and this was observed in the current study where amino acids metabolism intermediates such as L-valine and L-leucine were downregulated in the *T. zimbabwensis*-infected rats as compared to the control animals. These two branched chain amino acids, L-valine and L-leucine are essential amino acid molecules that are metabolized in muscles and offer energy (Brestenský et al., 2021).

After 21 days post infection, *T. zimbabwensis* larvae are expected to be fully lodged or located in the muscle of their host (Onkoba et al., 2016). In the present study, muscle larvae were first detected at day 28 dpi. The study conducted by Mukaratirwa et al. (2008) in baboons and monkeys reported modifications of muscle fiber architecture. Furthermore, the study observed basophilic transformation of muscle cells and swollen muscle fibers. Wu et al. (2009) reported that when *Trcichinella* new-born larvae invade host myofibres, they are subjected to inhospitable environment, therefore they modify it to suit their needs for survival. Wu et al. (2009) reported that after the larvae has invade the muscle cells, they grow and consume different kinds of nutrients including glucose. In our study, an essential metabolic pathway that was affected by *Trichinella* infection was the glycolysis/gluconeogenesis pathway and glucose and acetate biomarkers were found in this pathway. Glycolysis and gluconeogenesis are two essential pathways (Shah-Simpson et al., 2017) as carbon sources for anabolic synthesis (Xiong et al., 2011). In the present study, glucose was up-regulated in the *T. zimbabwensis*-infected animals, indicating that the production of glucose was not impeded by the infection. According to Shea-Donohue et al. (2017), in T cells and erythrocytes, nematode infection results in the upregulation of glucose transporter 1 (GLUT1). Moreover, Li et al. (2016) reported that *Trypanosoma cruzi* (an extra-cellular protozoa) infection increased host glucose transporter expression while Shah-Simpson et al. (2017) reported increased glucose uptake by the host cells and increased mitochondrial respiration during *Trypanosoma cruzi* infection.

During glycolysis, glucose is oxidized to form pyruvate, which is also converted to form acetyl-CoA that will enter the tricarboxylic acid cycle (TCA cycle). Furthermore, glucose is assumed to be burned by tissues *via* the TCA cycle, under aerobic condition (Hui et al., 2017). According to Montgomery et al. (2003), during *Trichinella spiralis* infection, there is higher levels of glucose uptake by the nurse cells as compared to the L6 cells. This can be related to the parasites ability to alter the host cells to accommodate their nutritional needs for long term survival in the host muscles. Therefore, this suggest that the cell and the enclosed parasite are metabolically active and require continuous supply of glucose. According to Montgomery and Stewart (1996), if the nurse cells have no glucose, the parasite will consume its glycogen reserves. The increase in glucose demand during *Trichinella* infection occurs simultaneously with the newborn larvae migration, penetration, establishment and encystment within the straited muscle (Wu et al., 2009). It has been hypothesized that high consumption of glucose by the rapidly growing parasite results in hypoglycemia (Wu et al., 2009). In addition, Wu et al. (2009) reported three possible causes of the hypoglycemia during *Trichinella* infection and these include high glucose consumption by the parasite, reduced absorptive capacity of the intestine, and impairment of glucose production by liver. The study by Sukhdeo and Mettrick (1984) reported that the reduced absorptive capacity of the intestine cannot be a leading cause, since it has been reported that during moderate infection, *Trichinella* infection does not affect glucose uptake by intestine. However, a study by Oltean et al. (2012) reported an increase in the blood glucose concentration of *Trichinella* spiralis-infected pigs 30 days post-infection comparative with uninfected ones. Therefore, this suggests that trichinellosis has an influence in glucose metabolism.

The potential biomarker that was associated with the TCA cycle was succinic acid which was downregulated in the infected animals compared to the controls. The TCA cycle also known as the Krebs cycle is essential for energy production as well as synthesis and degradation of biomolecules (Fleige et al., 2008). According to Akram (2014), the TCA cycle is a final common pathway for oxidation of protein, carbohydrates, and lipids. Amino acids play an essential role in the production of acetyl-CoA (Akram, 2014). After acetyl-CoA has entered the TCA cycle from glycolysis, it then reacts with oxaloacetate and form citrate. Furthermore, in a series of enzyme mediated reactions, succinate is produced which will then form oxaloacetate, starting the cycle again. In the present study, a prominent finding was the downregulation or depletion of the TCA cycle intermediate, succinate, in serum samples of *T. zimbabwensis*-infected rats when compared with the control rats. According to Akram (2014), acetyl-CoA is obtained from amino acids like, triacylglycerol, ketone bodies, isoleucine, lysine, leucine, and tyrosine, therefore, downregulation of metabolite leucine and tyrosine in the present study hindered the production of acetyl-CoA. Based on results from this study, it can be speculated that *Trichinella* infection obstructed energy production and the catabolism or breakdown of organic molecules in the infected rats.

In the present study, we have shown that using a non-targeted metabolomic approach, we were able to unravel different classes of metabolites that are associated with *T. zimbabwensis* infection in the rats. However, additional studies are needed to further verify the determined metabolites using a targeted metabolomics. The study discovered xenobiotics, which could be metabolites produced by the parasite itself, and therefore future studies should also explore the metabolic signature of the parasite. Serum samples from the study were collected following an invasive metabolomic approach hence, we recommend future studies in the exploration of use of noninvasive methods which include collection of stool and urine from host during infection.

## 5 Conclusion

These findings highlight the potential of metabolomics as a novel approach for fundamental investigations of host–pathogen interactions as well as for disease surveillance and control. The diagnostic accuracy of the discovered biomarkers combined have a strong discriminative power. In addition, the amino acids intermediates, L-tyrosine, histidine, and thiazole, due to their metabolic change and impact, may be the metabolites that have diagnostic potential for *T. zimbabwensis.*

## Data Availability

The original contributions presented in the study are included in the article/Supplementary Material, further inquiries can be directed to the corresponding author.
