# Supplementary material for: Preliminary insights on the metabolomics of Trichinella zimbabwensis infection in Sprague Dawley rats using GCxGC-TOF-MS (untargeted approach)
Source: Front Mol Biosci. 2023 Feb 17;10:1128542. doi: 10.3389/fmolb.2023.1128542 (PMC9983363; doi:10.3389/fmolb.2023.1128542)
Supplement: Supplementary file 1 [file Table1.DOCX]

Supplementary Material

Potential use of metabolomics as a diagnostic tool for *Trichi-nella zimbabwensis* infection in Sprague Dawley rats using GCxGC-TOF-MS (untargeted approach).

Innocent Syanda Ndlovu^1^ *, Ekuyikeno Silas^1^, Andre Vosloo, Selaelo Ivy Tshilwane^2^, Mamohale Chaisi^2,3^, and Samson Mukaratirwa^1,4^.

*** Correspondence:** Innocent Syanda Ndlovu: 213525571@stu.ukzn.ac.za

## Supplementary Figures


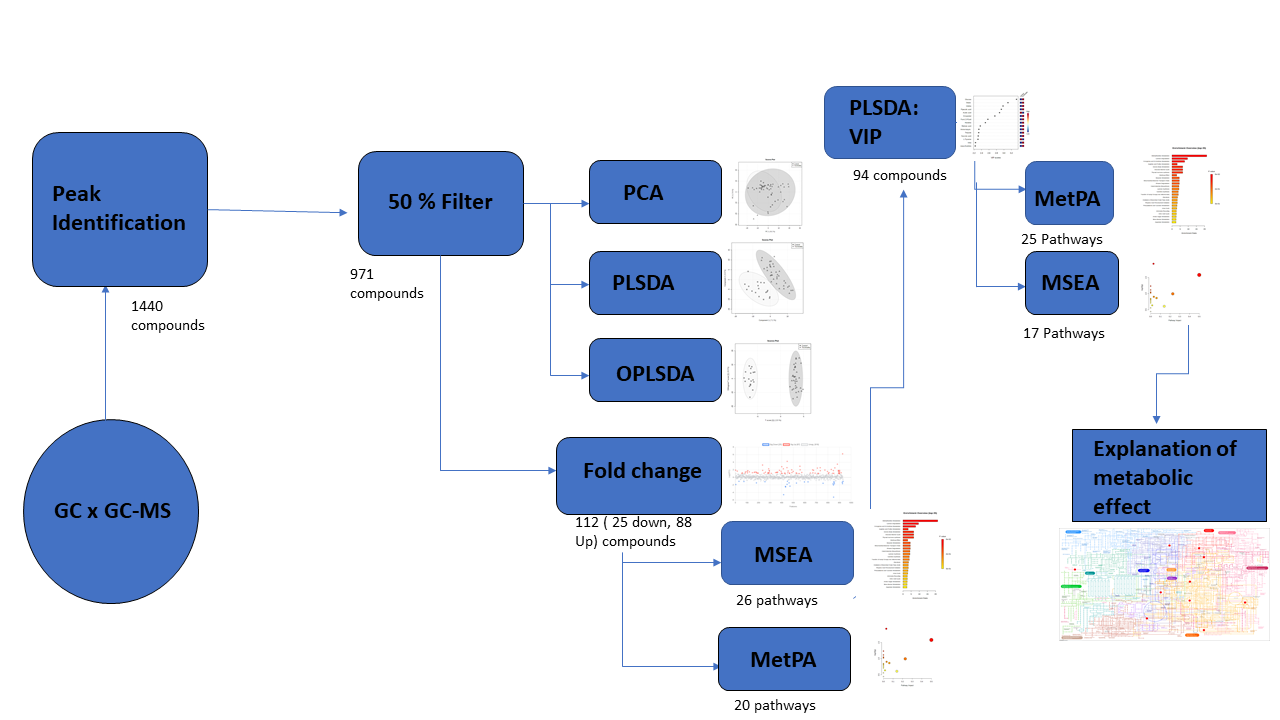


**Supplementary Figure S1**: Schematic diagram of procedures followed in the identification of compounds and pathway analysis.

**Additional file 2: Fig. S2**.


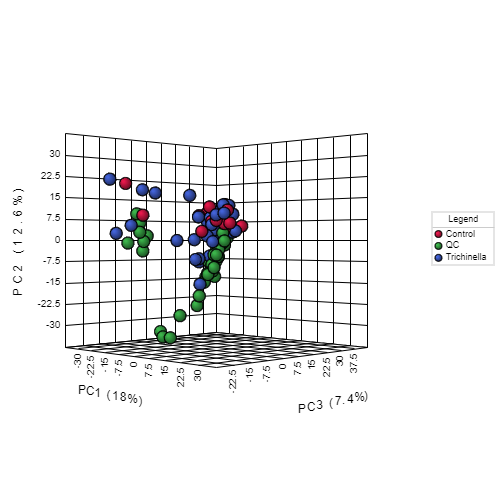


**Supplementary Figure S2**: Three-dimensional PCA scores plot showing the variation between the QC samples in relation to the variation between the serum samples. PCA, principal component analysis; QC, quality control.

**Additional file 3: Fig. S3.**


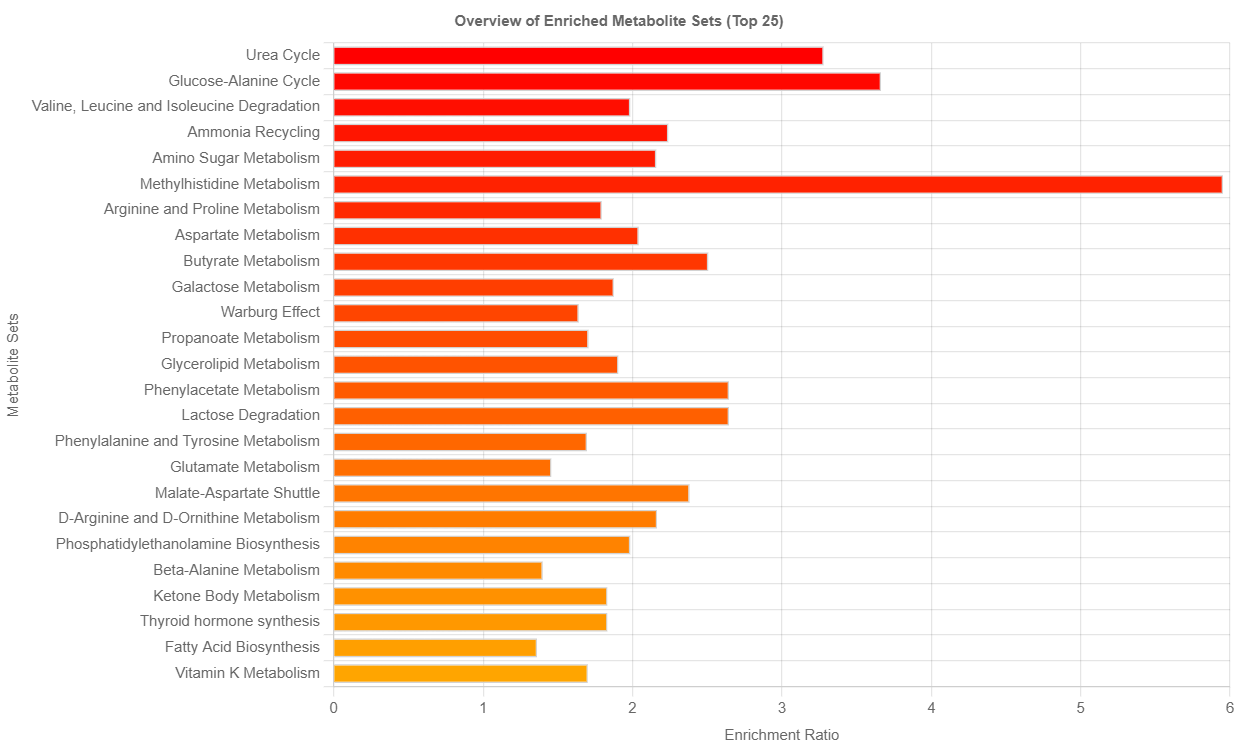


**A**


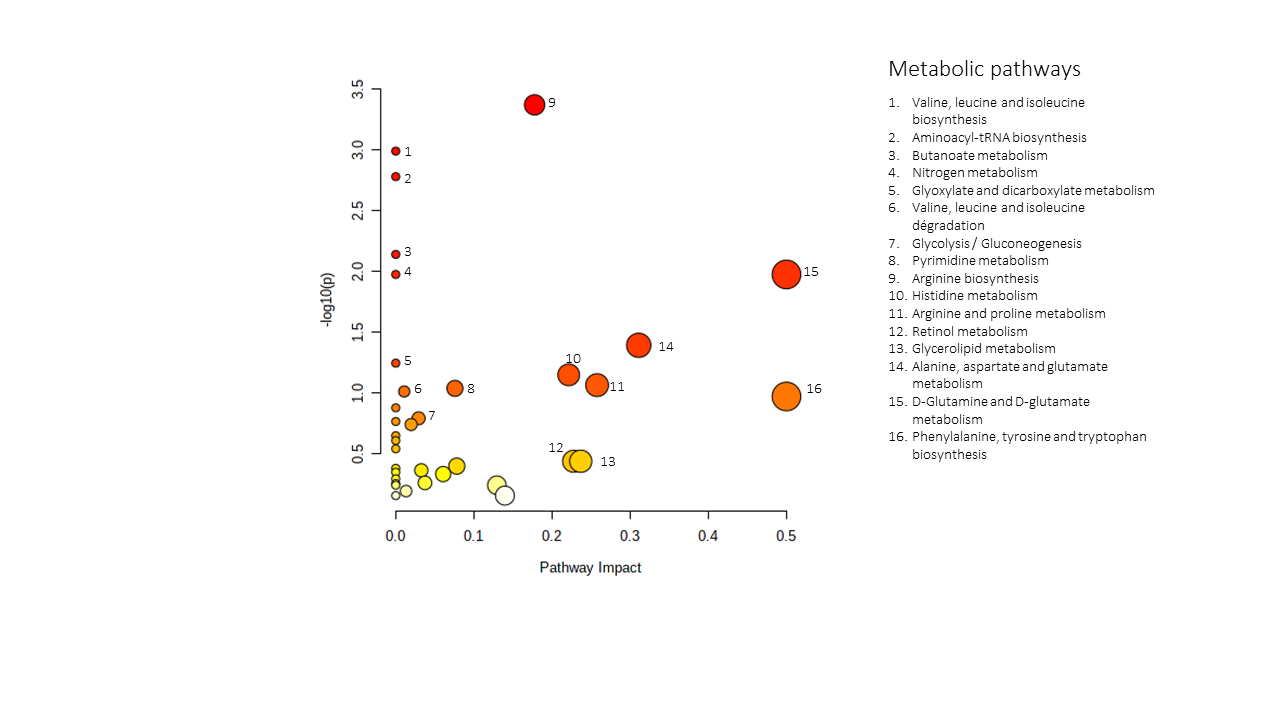


**B**

**Supplementary Figure S3**: Pathway analysis as generated by MetaboAnalyst software package. Identified metabolites and their relative quantity were used to calculate the enrichment and statistical significance. Metabolic pathways analysis associated with the 94 identified potential metabolites. A) Results of metabolite set enrichment analysis. The horizontal bars show a summary of metabolic pathways that were strongly affected or p in the Tz infected compared to the control group. B) Metabolic pathway analysis (MetPA). All the matched pathways are displayed as circles. The color and size of each circle are based on the p-value and pathway impact value, respectively. The most impacted pathways having high statistical significance scores are indicated with numbers.

**Additional file 4, figure S4**
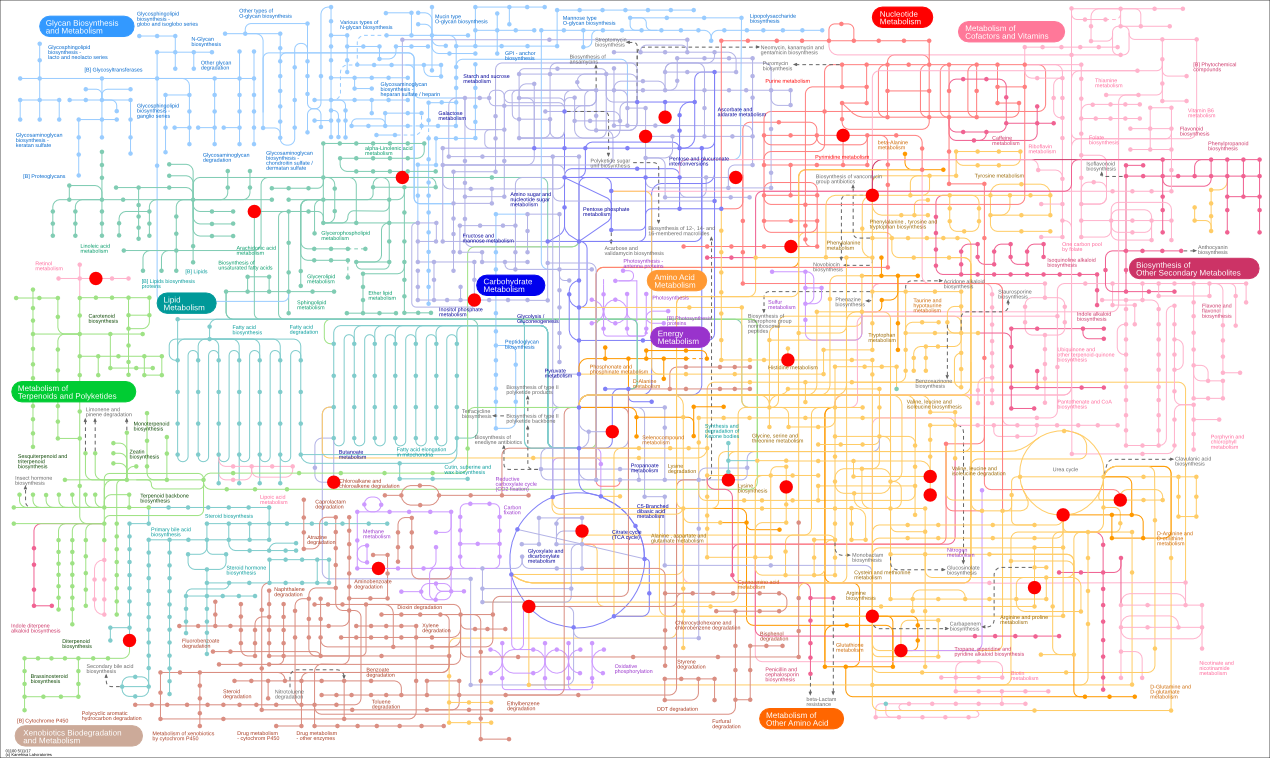
 **Supplementary Figure 4:** KEGG pathways map; the highlighted lines indicate the metabolic pathways associated with the all the identified differentially expressed metabolites markers. **Colored circles indicate the metabolic biomarkers.*

## Supplementary Tables

**Supplementary Tables**

**Additional file 1: Table S1.**

Supplementary Table S1:Key deferential metabolites of *Trichinella zimbabwensis* infection

| Different metabolites | VIP | Fold change | Variation Trend | Different metabolites | VIP | Fold change | Variation Trend |
| --- | --- | --- | --- | --- | --- | --- | --- |
| Glucose | 3.336 | 37.224 | ↑ | Trifluoroacetamide | 1,7687 | 0,48722 | ↓ |
| Silane | 3.108 | 3.699 | ↑ | Ribitol | 1,7585 | 0,48559 | ↓ |
| Uridine | 2.976 | 0,273 | ↓ | Butylmethylamine | 1,7507 | 0,4826 | ↓ |
| Pipecolic acid | 2.930 | 0,293 | ↓ | Propanetriol | 1,7444 | 0,47937 | ↓ |
| Acetic acid | 2.882 | 3.138 | ↑ | L-Valine | 1,7401 | 0,4785 | ↓ |
| D-mannitol | 2.757 | 2827 | ↑ | Scyllo-Inositol | 1,73 | 0,47801 | ↓ |
| Furo-2,3-Pyridine | 2.570 | 2556 | ↑ | Pipecolic acid | 1,7242 | 0,47747 | ↓ |
| Histidine | 2.503 | 0,457 | ↓ | Dimethoxy-2-methyl-2-butanol | 1,7238 | 0,47642 | ↓ |
| Retinoic acid | 2.367 | 0,471 | ↓ | Serine | 1,7234 | 0,47309 | ↓ |
| Amino-butyric acid | 2.328 | 0,892 | ↓ | Talofuranose | 1,7234 | 0,47177 | ↓ |
| Thiazole | 2.324 | 3871 | ↑ | Hexamethyl-3,6,8,10,12,15-hexaoxa | 1,7219 | 0,46798 | ↓ |
| Succinic acid | 2.311 | 2821 | ↑ | Oxyproline | 1,7189 | 0,46753 | ↓ |
| L-Tyrosine | 2.304 | 0,822 | ↓ | Ribitol | 1,7166 | 0,46726 | ↓ |
| Urea | 2.238 | 8810 | ↑ | Campesterol | 1,6904 | 0,46341 | ↓ |
| meso-Erythritol | 2,152 | 40,236 | ↑ | Isopropyl amine | 1,6847 | 0,46338 | ↓ |
| Oxo butyric acid | 2,149 | 8,7875 | ↑ | Triazolo | 1,6825 | 0,46138 | ↓ |
| Silanol | 2,142 | 6,6931 | ↑ | Pentafluoro phenol | 1,6621 | 0,45323 | ↓ |
| 1H-Indene, 1-hexadecyl-2,3-dihydro | 2,121 | 5,5302 | ↑ | Pyrrolidine Dione | 1,6503 | 0,4436 | ↓ |
| L-Hydroxyproline | 2,111 | 5,1567 | ↑ | Pyridine | 1,65 | 0,44354 | ↓ |
| Glycerin acid | 2,106 | 5,0063 | ↑ | Propanoic acid | 1,6353 | 0,44007 | ↓ |
| Thymidine | 2,102 | 4,9982 | ↑ | Hexamethylene glycol | 1,6235 | 0,4396 | ↓ |
| Methoxy acetic acid | 2,068 | 4,5303 | ↑ | Acetate | 1,6059 | 0,43321 | ↓ |
| 5-Nonanone, oxime | 2,063 | 4,1438 | ↑ | Xylopyranose | 1,6053 | 0,43152 | ↓ |
| 2-(N-Morpholinyl)ethylphosphine | 2,057 | 3,8551 | ↑ | Nitrobarbiturate | 1,5976 | 0,42957 | ↓ |
| Silane, tetramethyl- UM 158 | 2,033 | 3,5359 | ↑ | Cycloserine | 1,5968 | 0,41723 | ↓ |
| Penta siloxane | 2,023 | 3,4131 | ↑ | Hydroxyquinoline | 1,5954 | 0,41575 | ↓ |
| d-Galactose | 2,015 | 3,2248 | ↑ | Ethylene glycol | 1,5945 | 0,4145 | ↓ |
| Trifluoro acetamidine | 1,507 | 3,1866 | ↑ | 2-(Ethylamino)ethanol, | 1,5009 | 0,40983 | ↓ |
| L-Glutamic acid | 1,501 | 3,0621 | ↑ | Aminobutanoic acid | 1,5041 | 0,40616 | ↓ |
| Propanoate | 1,500 | 2,8725 | ↑ | Amino-2-piperidone | 1,5025 | 0,40535 | ↓ |
| á-Alanine | 1,945 | 2,8284 | ↑ | L-Leucine | 1,5044 | 0,40309 | ↓ |
| d-Galactose-o-methyloxyme | 1,936 | 2,8106 | ↑ | Furo[2,3-c]pyridine | 1,5023 | 0,40133 | ↓ |
| 2-Pyridinemethanol | 1,933 | 2,6655 | ↑ | Pentanoic acid | 1,5022 | 0,39791 | ↓ |
| 4-Pyrimidinamine | 1,931 | 2,6484 | ↑ | Tricarballylic acid | 1,5044 | 0,39714 | ↓ |
| Imidazole | 1,891 | 2,5663 | ↑ | Glycerol | 1,5228 | 0,39528 | ↓ |
| Ethanamide | 1,890 | 2,4676 | ↑ | (2,5-Dimethoxy-phenyl)-phenyl-methanol UM | 1,501 | 0,38605 | ↓ |
| á-Hydroxypyruvic acid | 1,865 | 2,4631 | ↑ | Ketoisocaproic acid | 1,50245 | 0,38265 | ↓ |
| Dimethyl tartarate | 1,850 | 2,2604 | ↑ | Amphetamine | 1,5024 | 0,38235 | ↓ |
| Nitrobarbiturate | 1,847 | 2,2371 | ↑ | Butanoic acid | 1,5046 | 0,38108 | ↓ |
| Octenoic acid | 1,821 | 2,2155 | ↑ | Oxazole | 1,5042 | 0,37017 | ↓ |
| Formamidopyrimidin | 1,808 | 2,2025 | ↑ | Chloromethyl cyanide | 1,5043 | 0,36961 | ↓ |
| Dichlorobenzoate | 1,799 | 2,1293 | ↑ | Pantothenic-Acid | 1,5022 | 0,36938 | ↓ |
| Ethanolamine | 1,796 | 2,0957 | ↑ | Trimethyl-1-pentanol | 1,503 | 0,36895 | ↓ |
| Silane, 1,3-propanediylbis | 1,790 | 2,0818 | ↑ | DL-Glyceraldehyde | 1,5 | 0,35844 | ↓ |
| L-Glutamine | 1,783 | 0,49699 | ↓ | n-Butylamine | 1,5007 | 0,35486 | ↓ |
| Methanol UM 102 FLAG | 1,780 | 0,49298 | ↓ | Hexanedioic acid | 1,5007 | 0,34759 | ↓ |
| Octanoic acid | 1,777 | 0,49246 | ↓ | Oxathiane | 1,5072 | 0,34643 | ↓ |
| L-Ornithine | 1,504 | 0,34035 | ↓ |  |  |  |  |
|  | | | |  | | |  |

Note:"↑" = compound is up-regulated and "↓" = compound is down-regulated

Additional file 2: Table. S2:

Supplementary Table S2: Illustration of Fisher’s Least Significant Difference (LSD) post hoc multivariate analysis of metabolites difference in different days post *Trichinella* infection.

| Metabolites | f.value | p.value | Fisher's LSD |
| --- | --- | --- | --- |
| Glucose | 21.093 | 4.2914E-11 | Day 14 - Day 0; Day 21 - Day 0; Day 28 - Day 0; Day 0 - Day 35; Day 7 - Day 0; Day 14 - Day 21; Day 14 - Day 35; Day 7 - Day 14; Day 21 - Day 35; Day 7 - Day 21; Day 28 - Day 35; Day 7 - Day 28; Day 7 - Day 35 |
| Silane | 13.103 | 4.667E-08 | Day 0 - Day 14; Day 0 - Day 21; Day 0 - Day 28; Day 0 - Day 35; Day 0 - Day 7; Day 21 - Day 14; Day 28 - Day 14; Day 35 - Day 14; Day 7 - Day 14 |
| Uridine | 11.504 | 2.4885E-07 | Day 0 - Day 14; Day 0 - Day 21; Day 0 - Day 28; Day 0 - Day 7; Day 21 - Day 14; Day 28 - Day 14; Day 35 - Day 14; Day 35 - Day 21; Day 35 - Day 28; Day 35 - Day 7 |
| Pipecolic acid | 10.976 | 4.4314E-07 | Day 14 - Day 0; Day 21 - Day 0; Day 7 - Day 0; Day 14 - Day 21; Day 14 - Day 28; Day 14 - Day 35; Day 21 - Day 35; Day 28 - Day 35; Day 7 - Day 28; Day 7 - Day 35 |
| Acetic acid | 9.6997 | 1.8982E-06 | Day 21 - Day 0; Day 28 - Day 0; Day 7 - Day 0; Day 21 - Day 14; Day 28 - Day 14; Day 14 - Day 35; Day 7 - Day 14; Day 21 - Day 35; Day 28 - Day 35; Day 7 - Day 35 |
| D-mannitol | 9.6725 | 1.9598E-06 | Day 0 - Day 28; Day 0 - Day 35; Day 14 - Day 28; Day 14 - Day 35; Day 21 - Day 28; Day 21 - Day 35; Day 7 - Day 28; Day 7 - Day 35 |
| Furo-2,3-Pyridine | 9.0207 | 4.2617E-06 | Day 14 - Day 0; Day 7 - Day 0; Day 14 - Day 21; Day 14 - Day 28; Day 14 - Day 35; Day 14 - Day 7; Day 7 - Day 21; Day 7 - Day 28; Day 7 - Day 35 |
| Histidine | 8.5121 | 7.9451E-06 | Day 0 - Day 14; Day 0 - Day 21; Day 0 - Day 28; Day 0 - Day 35; Day 0 - Day 7; Day 28 - Day 14; Day 35 - Day 14; Day 7 - Day 14; Day 7 - Day 21 |
| Retinoic acid | 8.3015 | 0.000010328 | Day 14 - Day 0; Day 21 - Day 0; Day 28 - Day 0; Day 7 - Day 0; Day 14 - Day 28; Day 14 - Day 35; Day 21 - Day 35; Day 7 - Day 28; Day 7 - Day 35 |
| Amino-butyric acid | 7.4278 | 0.00003156 | Day 0 - Day 21; Day 0 - Day 35; Day 14 - Day 21; Day 14 - Day 35; Day 28 - Day 21; Day 7 - Day 21; Day 28 - Day 35; Day 7 - Day 28; Day 7 - Day 35 |
| Thiazole | 7.0666 | 0.000050795 | Day 14 - Day 0; Day 0 - Day 28; Day 7 - Day 0; Day 14 - Day 21; Day 14 - Day 28; Day 21 - Day 28; Day 7 - Day 21; Day 35 - Day 28; Day 7 - Day 28 |
| Succinic acid | 6.8289 | 0.000069784 | Day 14 - Day 0; Day 21 - Day 0; Day 35 - Day 0; Day 7 - Day 0; Day 14 - Day 28; Day 21 - Day 28; Day 35 - Day 28; Day 7 - Day 28 |
| L-Tyrosine | 6.4164 | 0.00012221 | Day 0 - Day 28; Day 14 - Day 21; Day 14 - Day 28; Day 7 - Day 21; Day 35 - Day 28; Day 7 - Day 28 |
| Urea | 6.4096 | 0.00012335 | Day 0 - Day 35; Day 14 - Day 35; Day 21 - Day 35; Day 28 - Day 35; Day 7 - Day 35 |
| Meso-Erythritol | 6.2617 | 0.00015122 | Day 14 - Day 0; Day 21 - Day 0; Day 28 - Day 0; Day 7 - Day 0; Day 14 - Day 35; Day 21 - Day 35; Day 28 - Day 35; Day 7 - Day 35 |

**Additional file 3: Table S3**

**Supplementary Table S3**: Metabolic pathways for MetPA and metabolite involved in each pathway based on the 94 identified potential metabolite markers.

| Pathway Name | Match Status | Metabolite | p-value | FDR | Impact |
| --- | --- | --- | --- | --- | --- |
| [Arginine biosynthesis](https://dev.metaboanalyst.ca/MetaboAnalyst/Secure/pathway/ResultView.xhtml) | [4/14](https://dev.metaboanalyst.ca/MetaboAnalyst/Secure/pathway/ResultView.xhtml) | L-Glutamate, L-Ornithine, L-Glutamine; Urea | 4.2469E-4 | 0.035674 | 0.17766 |
| [Valine, leucine and isoleucine biosynthesis](https://dev.metaboanalyst.ca/MetaboAnalyst/Secure/pathway/ResultView.xhtml) | [3/8](https://dev.metaboanalyst.ca/MetaboAnalyst/Secure/pathway/ResultView.xhtml) | L-Leucine; 4-Methyl-2-oxopentanoate; L-Valine | 0.0010199 | 0.042836 | 0.0 |
| [Aminoacyl-tRNA biosynthesis](https://dev.metaboanalyst.ca/MetaboAnalyst/Secure/pathway/ResultView.xhtml) | [6/48](https://dev.metaboanalyst.ca/MetaboAnalyst/Secure/pathway/ResultView.xhtml) | L-Histidine; L-Glutamine; L-Valine; L-Leucine; L-Tyrosine; L-Glutamate | 0.0016557 | 0.04636 | 0.0 |
| [Butanoate metabolism](https://dev.metaboanalyst.ca/MetaboAnalyst/Secure/pathway/ResultView.xhtml) | [3/15](https://dev.metaboanalyst.ca/MetaboAnalyst/Secure/pathway/ResultView.xhtml) | L-Glutamate; Butanoic acid; Succinate; | 0.0072302 | 0.14799 | 0.0 |
| [Nitrogen metabolism](https://dev.metaboanalyst.ca/MetaboAnalyst/Secure/pathway/ResultView.xhtml) | [2/6](https://dev.metaboanalyst.ca/MetaboAnalyst/Secure/pathway/ResultView.xhtml) | L-Glutamate; L-Glutamine | 0.010571 | 0.14799 | 0.0 |
| [D-Glutamine and D-glutamate metabolism](https://dev.metaboanalyst.ca/MetaboAnalyst/Secure/pathway/ResultView.xhtml) | [2/6](https://dev.metaboanalyst.ca/MetaboAnalyst/Secure/pathway/ResultView.xhtml) | L-Glutamate; L-Glutamine | 0.010571 | 0.14799 | 0.5 |
| [Alanine, aspartate and glutamate metabolism](https://dev.metaboanalyst.ca/MetaboAnalyst/Secure/pathway/ResultView.xhtml) | [3/28](https://dev.metaboanalyst.ca/MetaboAnalyst/Secure/pathway/ResultView.xhtml) | L-Glutamate; L-Glutamate, Succinate | 0.040505 | 0.48605 | 0.3109 |
| [Glyoxylate and dicarboxylate metabolism](https://dev.metaboanalyst.ca/MetaboAnalyst/Secure/pathway/ResultView.xhtml) | [3/32](https://dev.metaboanalyst.ca/MetaboAnalyst/Secure/pathway/ResultView.xhtml) | L-Glutamate; Acetate; L-Glutamine | 0.056806 | 0.59647 | 0.0 |
| [Histidine metabolism](https://dev.metaboanalyst.ca/MetaboAnalyst/Secure/pathway/ResultView.xhtml) | [2/16](https://dev.metaboanalyst.ca/MetaboAnalyst/Secure/pathway/ResultView.xhtml) | L-Glutamate; L-Histidine | 0.070935 | 0.66206 | 0.22131 |
| [Arginine and proline metabolism](https://dev.metaboanalyst.ca/MetaboAnalyst/Secure/pathway/ResultView.xhtml) | [3/38](https://dev.metaboanalyst.ca/MetaboAnalyst/Secure/pathway/ResultView.xhtml) | Hydroxyproline; L-Glutamate; L-Ornithine | 0.08619 | 0.68033 | 0.25762 |
| [Pyrimidine metabolism](https://dev.metaboanalyst.ca/MetaboAnalyst/Secure/pathway/ResultView.xhtml) | [3/39](https://dev.metaboanalyst.ca/MetaboAnalyst/Secure/pathway/ResultView.xhtml) | L-Glutamine; Uridine; Thymidine | 0.091619 | 0.68033 | 0.0757 |
| [Valine, leucine, and isoleucine degradation](https://dev.metaboanalyst.ca/MetaboAnalyst/Secure/pathway/ResultView.xhtml) | [3/40](https://dev.metaboanalyst.ca/MetaboAnalyst/Secure/pathway/ResultView.xhtml) | L-Valine; 4-Methyl-2-oxopentanoate; L-Leucine | 0.097189 | 0.68033 | 0.01084 |
| [Phenylalanine, tyrosine, and tryptophan biosynthesis](https://dev.metaboanalyst.ca/MetaboAnalyst/Secure/pathway/ResultView.xhtml) | [1/4](https://dev.metaboanalyst.ca/MetaboAnalyst/Secure/pathway/ResultView.xhtml) | L-Tyrosine | 0.10687 | 0.69055 | 0.5 |
| [Propanoate metabolism](https://dev.metaboanalyst.ca/MetaboAnalyst/Secure/pathway/ResultView.xhtml) | [2/23](https://dev.metaboanalyst.ca/MetaboAnalyst/Secure/pathway/ResultView.xhtml) | Succinate; Propanoate | 0.13251 | 0.79507 | 0.0 |
| [Glycolysis / Gluconeogenesis](https://dev.metaboanalyst.ca/MetaboAnalyst/Secure/pathway/ResultView.xhtml) | [2/26](https://dev.metaboanalyst.ca/MetaboAnalyst/Secure/pathway/ResultView.xhtml) | beta-D-Glucose; Acetate | 0.16171 | 0.89824 | 0.02927 |
| [Galactose metabolism](https://dev.metaboanalyst.ca/MetaboAnalyst/Secure/pathway/ResultView.xhtml) | [2/27](https://dev.metaboanalyst.ca/MetaboAnalyst/Secure/pathway/ResultView.xhtml) | Glycerol; myo-Inositol | 0.1717 | 0.89824 | 0.0 |
| [Glutathione metabolism](https://dev.metaboanalyst.ca/MetaboAnalyst/Secure/pathway/ResultView.xhtml) | [2/28](https://dev.metaboanalyst.ca/MetaboAnalyst/Secure/pathway/ResultView.xhtml) | L-Glutamate; L-Ornithine | 0.18179 | 0.89824 | 0.01966 |
| [Ubiquinone and other terpenoid-quinone biosynthesis](https://dev.metaboanalyst.ca/MetaboAnalyst/Secure/pathway/ResultView.xhtml) | [1/9](https://dev.metaboanalyst.ca/MetaboAnalyst/Secure/pathway/ResultView.xhtml) | L-Tyrosine | 0.22488 | 1.0 | 0.0 |
| [Ascorbate and aldarate metabolism](https://dev.metaboanalyst.ca/MetaboAnalyst/Secure/pathway/ResultView.xhtml) | [1/10](https://dev.metaboanalyst.ca/MetaboAnalyst/Secure/pathway/ResultView.xhtml) | L-Tyrosine | 0.24658 | 1.0 | 0.0 |
| [Phenylalanine metabolism](https://dev.metaboanalyst.ca/MetaboAnalyst/Secure/pathway/ResultView.xhtml) | [1/12](https://dev.metaboanalyst.ca/MetaboAnalyst/Secure/pathway/ResultView.xhtml) | L-Tyrosine | 0.28822 | 1.0 | 0.0 |
| [Retinol metabolism](https://dev.metaboanalyst.ca/MetaboAnalyst/Secure/pathway/ResultView.xhtml) | [1/16](https://dev.metaboanalyst.ca/MetaboAnalyst/Secure/pathway/ResultView.xhtml) | Retinoate | 0.36488 | 1.0 | 0.22754 |
| [Glycolipid metabolism](https://dev.metaboanalyst.ca/MetaboAnalyst/Secure/pathway/ResultView.xhtml) | [1/16](https://dev.metaboanalyst.ca/MetaboAnalyst/Secure/pathway/ResultView.xhtml) | Glycerol | 0.36488 | 1.0 | 0.23676 |
| [Pentose and glucuronate interconversions](https://dev.metaboanalyst.ca/MetaboAnalyst/Secure/pathway/ResultView.xhtml) | [1/18](https://dev.metaboanalyst.ca/MetaboAnalyst/Secure/pathway/ResultView.xhtml) | D-Xylose | 0.40012 | 1.0 | 0.07812 |
| [Pantothenate and CoA biosynthesis](https://dev.metaboanalyst.ca/MetaboAnalyst/Secure/pathway/ResultView.xhtml) | [1/19](https://dev.metaboanalyst.ca/MetaboAnalyst/Secure/pathway/ResultView.xhtml) | L-Valine | 0.41702 | 1.0 | 0.0 |
| [Citrate cycle (TCA cycle)](https://dev.metaboanalyst.ca/MetaboAnalyst/Secure/pathway/ResultView.xhtml) | [1/20](https://dev.metaboanalyst.ca/MetaboAnalyst/Secure/pathway/ResultView.xhtml) | Succinate | 0.43345 | 1.0 | 0.03273 |
| [beta-Alanine metabolism](https://dev.metaboanalyst.ca/MetaboAnalyst/Secure/pathway/ResultView.xhtml) | [1/21](https://dev.metaboanalyst.ca/MetaboAnalyst/Secure/pathway/ResultView.xhtml) | L-Histidine | 0.44943 | 1.0 | 0.0 |
| [Pyruvate metabolism](https://dev.metaboanalyst.ca/MetaboAnalyst/Secure/pathway/ResultView.xhtml) | [1/22](https://dev.metaboanalyst.ca/MetaboAnalyst/Secure/pathway/ResultView.xhtml) | Acetate | 0.46497 | 1.0 | 0.06065 |
| [Lysine degradation](https://dev.metaboanalyst.ca/MetaboAnalyst/Secure/pathway/ResultView.xhtml) | [1/25](https://dev.metaboanalyst.ca/MetaboAnalyst/Secure/pathway/ResultView.xhtml) | L-Pipecolate | 0.50907 | 1.0 | 0.0 |
| [Phosphatidylinositol signaling system](https://dev.metaboanalyst.ca/MetaboAnalyst/Secure/pathway/ResultView.xhtml) | [1/28](https://dev.metaboanalyst.ca/MetaboAnalyst/Secure/pathway/ResultView.xhtml) | myo-Inositol | 0.54961 | 1.0 | 0.03736 |
| [Purine metabolism](https://dev.metaboanalyst.ca/MetaboAnalyst/Secure/pathway/ResultView.xhtml) | [2/66](https://dev.metaboanalyst.ca/MetaboAnalyst/Secure/pathway/ResultView.xhtml) | L-Glutamine; Urea | 0.55691 | 1.0 | 0.0 |
| [Porphyrin and chlorophyll metabolism](https://dev.metaboanalyst.ca/MetaboAnalyst/Secure/pathway/ResultView.xhtml) | [1/30](https://dev.metaboanalyst.ca/MetaboAnalyst/Secure/pathway/ResultView.xhtml) | L-Glutamate | 0.5748 | 1.0 | 0.0 |
| [Inositol phosphate metabolism](https://dev.metaboanalyst.ca/MetaboAnalyst/Secure/pathway/ResultView.xhtml) | [1/30](https://dev.metaboanalyst.ca/MetaboAnalyst/Secure/pathway/ResultView.xhtml) | myo-Inositol | 0.5748 | 1.0 | 0.12939 |
| [Glycerophospholipid metabolism](https://dev.metaboanalyst.ca/MetaboAnalyst/Secure/pathway/ResultView.xhtml) | [1/36](https://dev.metaboanalyst.ca/MetaboAnalyst/Secure/pathway/ResultView.xhtml) | Ethanolamine | 0.6424 | 1.0 | 0.01324 |
| [Steroid biosynthesis](https://dev.metaboanalyst.ca/MetaboAnalyst/Secure/pathway/ResultView.xhtml) | [1/42](https://dev.metaboanalyst.ca/MetaboAnalyst/Secure/pathway/ResultView.xhtml) | Campesterol | 0.69947 | 1.0 | 0.0 |
| [Tyrosine metabolism](https://dev.metaboanalyst.ca/MetaboAnalyst/Secure/pathway/ResultView.xhtml) | [1/42](https://dev.metaboanalyst.ca/MetaboAnalyst/Secure/pathway/ResultView.xhtml) | L-Tyrosine; | 0.69947 | 1.0 | 0.13972 |
