# Supplementary material for: Preliminary insights on the metabolomics of Trichinella zimbabwensis infection in Sprague Dawley rats using GCxGC-TOF-MS (untargeted approach)
Source: Front Mol Biosci. 2023 Feb 17;10:1128542. doi: 10.3389/fmolb.2023.1128542 (PMC9983363; doi:10.3389/fmolb.2023.1128542)
Supplement: Supplementary file 2 [file Table4.DOCX]

**Manuscript figures.**


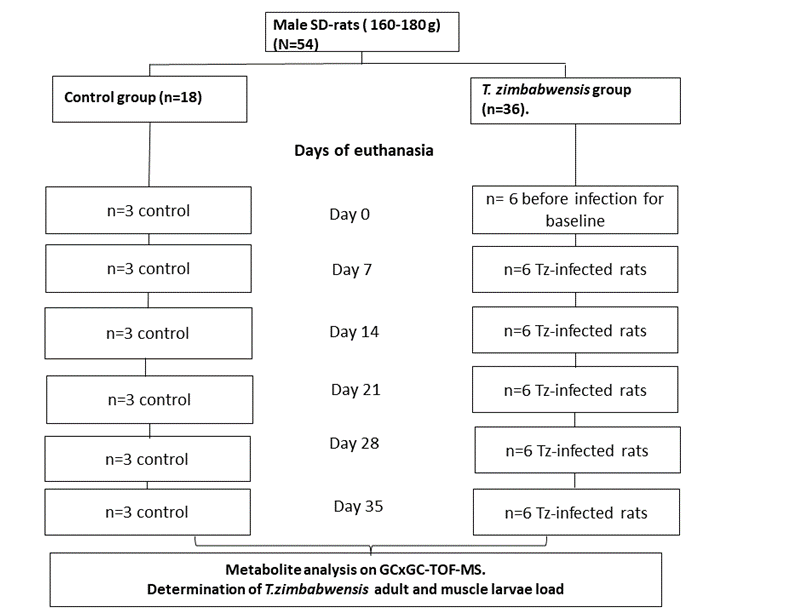


**Figure 1**: Schematic diagram of the experimental design. SD = Sprague-Dawley; *T. zimbabwensis = Trichinella zimbabwensis,* T.z -infected=*Trichinella zimbabwensis* infected.

**Figure 2:** Mean number (± SD) of intestinal adult worms and muscle larval counts recovered from Sprague Dawley rats infected with *T. zimbabwensis.* AW = Adult worms; ML = Muscle larvae.


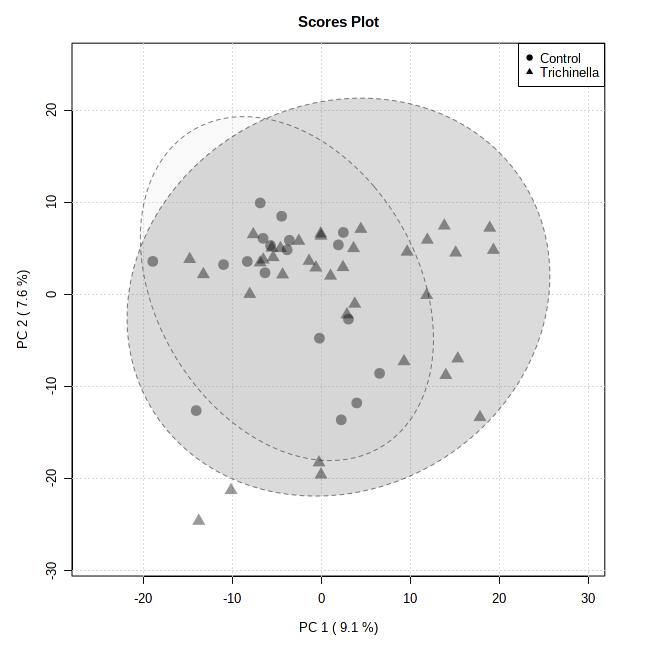

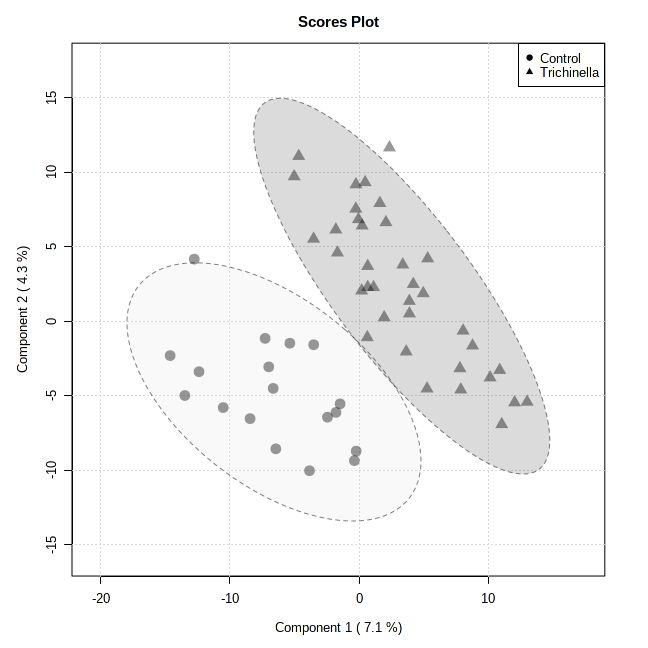

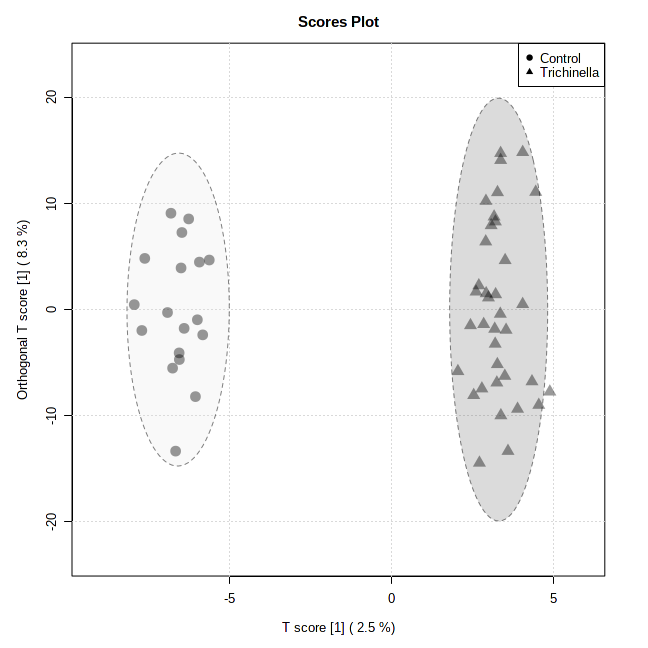


**C**

**B**

**A**

**Figure 3**: Principal Component Analysis (PCA) (A), PLS Discriminant Analysis (PLS-DA) (B), and (C) Orthogonal PLS-DA score plots. The score plots, the abscissa PC1, and the ordinate PC2 represent the scores of the principal components ranking the first and the second, respectively, and different shapes of the scattered points represent the different groups of the samples. The explained variances are shown in brackets. The Orthogonal PLS-DA score plot (c) of Tz-infected rats (Grey triangles) and the control rats (Grey circles).

*
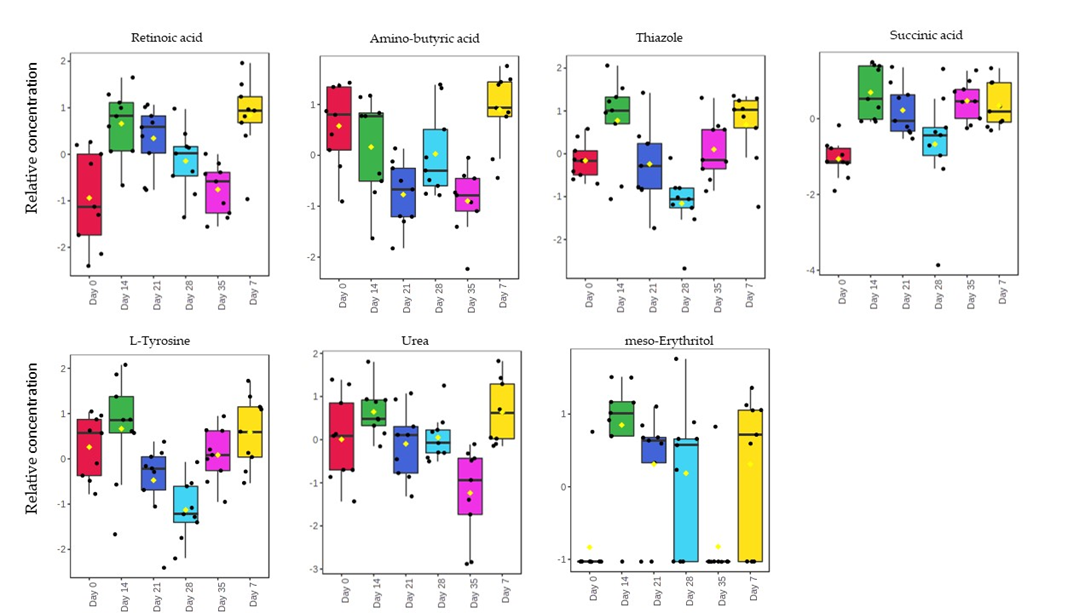
*
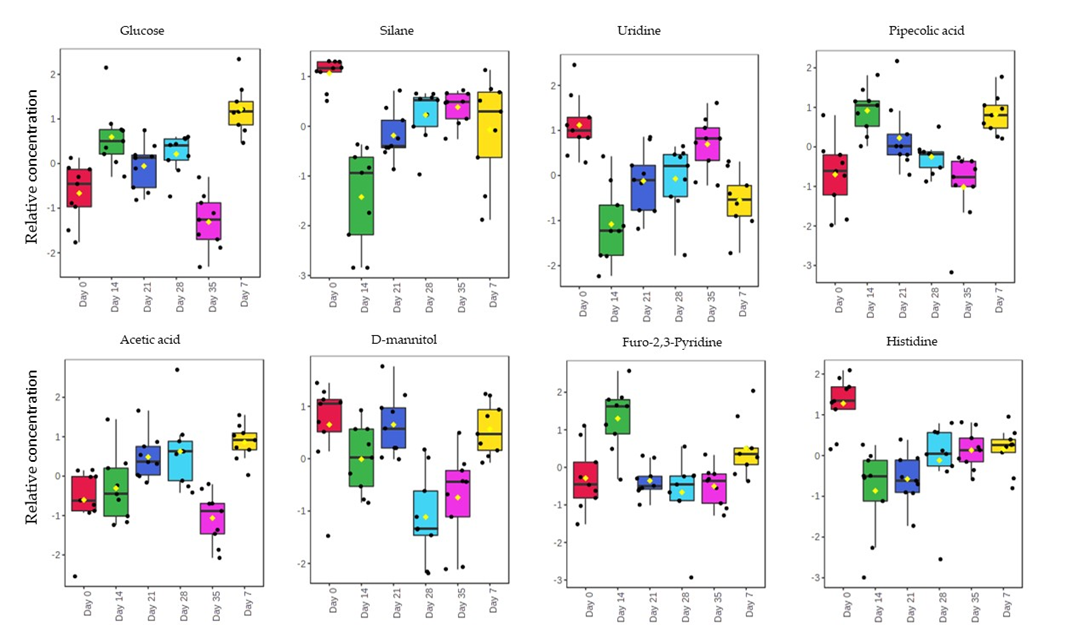
**Figure 4**: Box-Whisker plot for the significantly different metabolites (p-value ≤ 0.05). The top 15 significantly different metabolites identified and their relative concentrations of each plotted against the different days post infection.


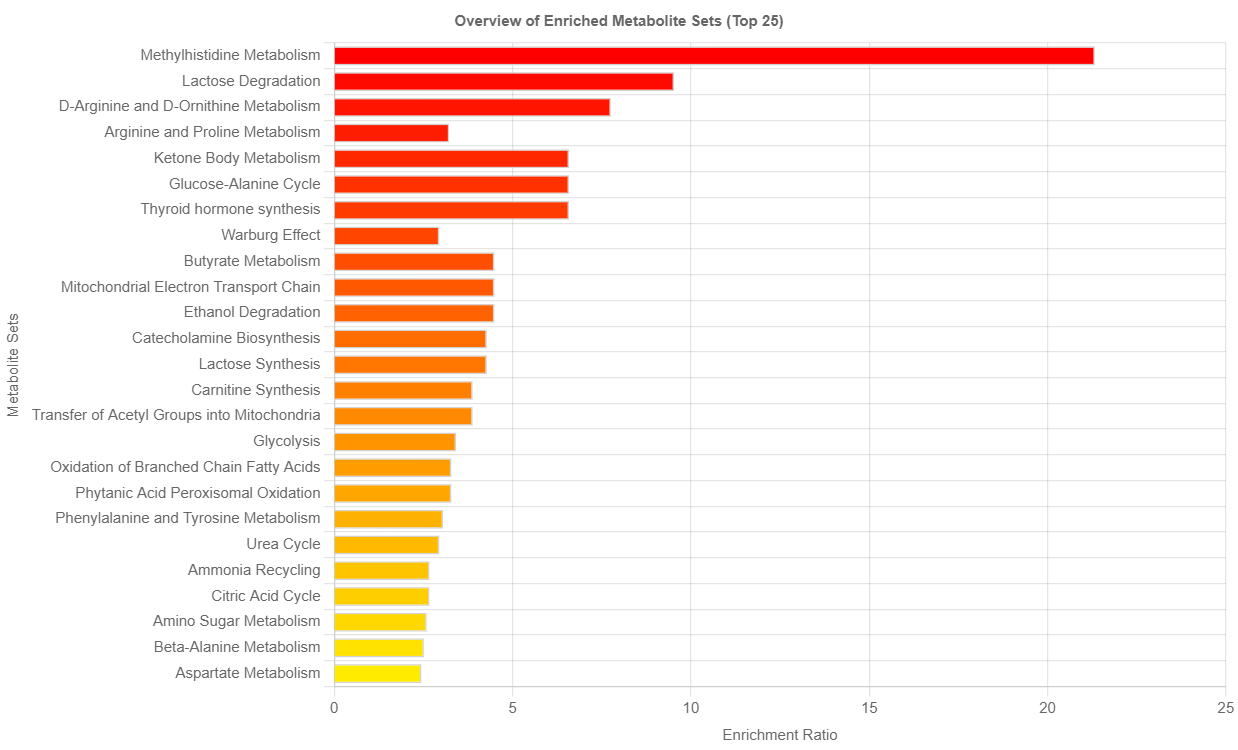


**Figure 5**: Metabolic pathways associated with the top 15 identified potential metabo-lites. The horizontal bars show a summary of metabolic pathways that were strongly affected in the *Trichinella zimbabwensis* infected group compared to the control. Color intensity (white to red) reflects increasing statistical significance.

**
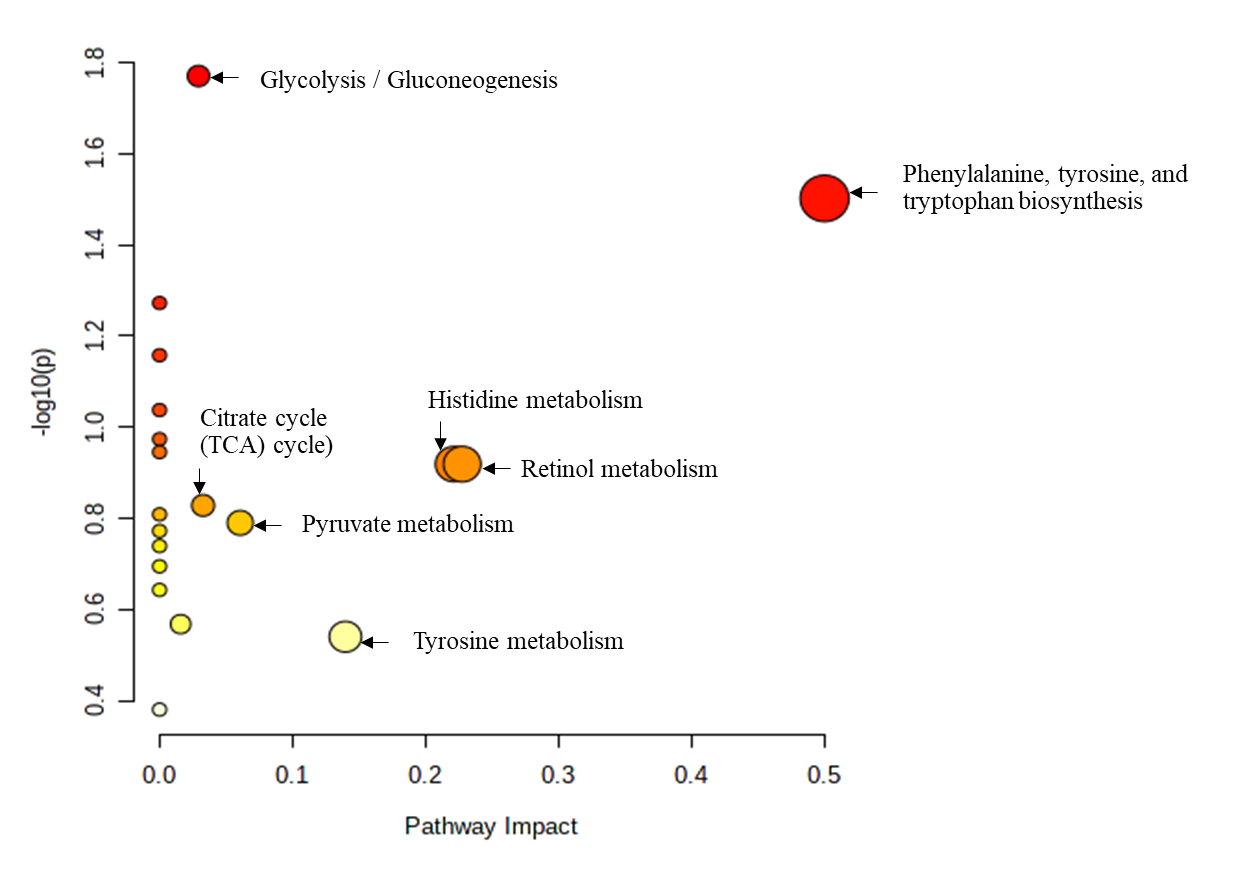
**

**Figure 6**: Metabolic pathways associated with the top 15 identified metabolites mark-ers. All the matched pathways are displayed as circles. The node size is proportional to the enrichment ratio. Light yellow to red indicates the p-value from small to large. The color and size of each circle are based on the p-value and pathway impact value, re-spectively. The most impacted pathways having high statistical significance scores are indicated with their names.


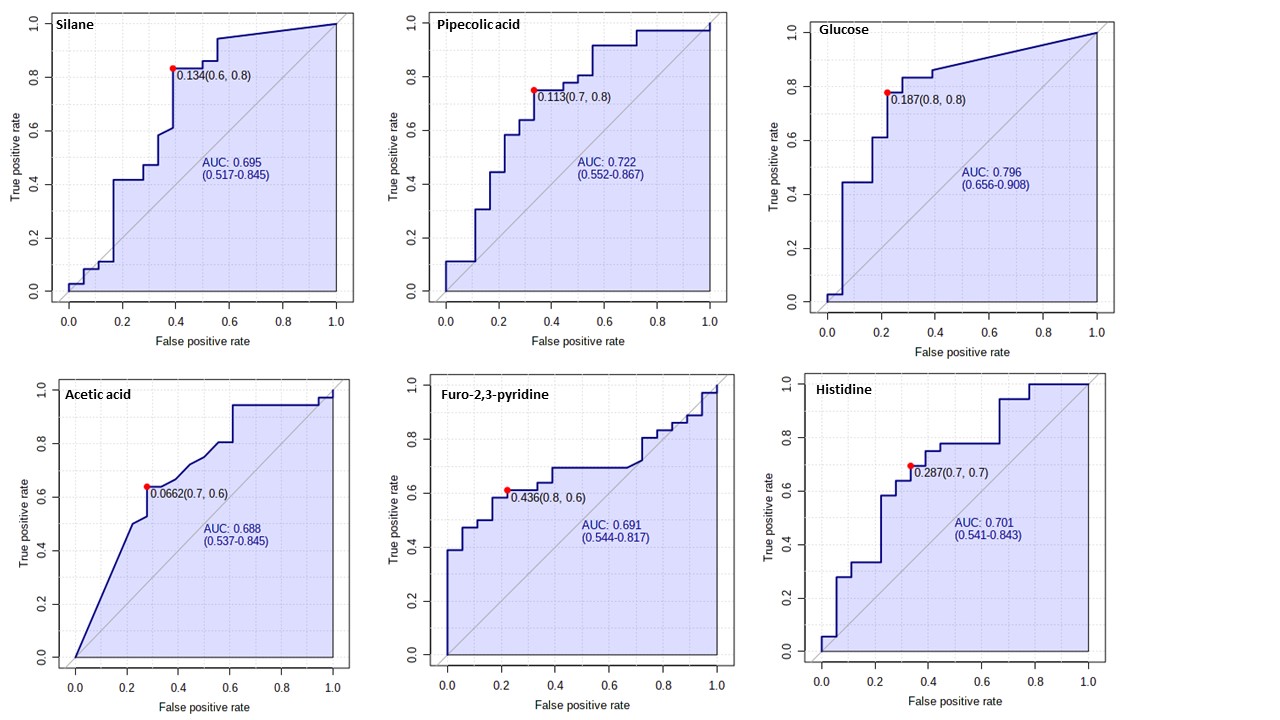
***
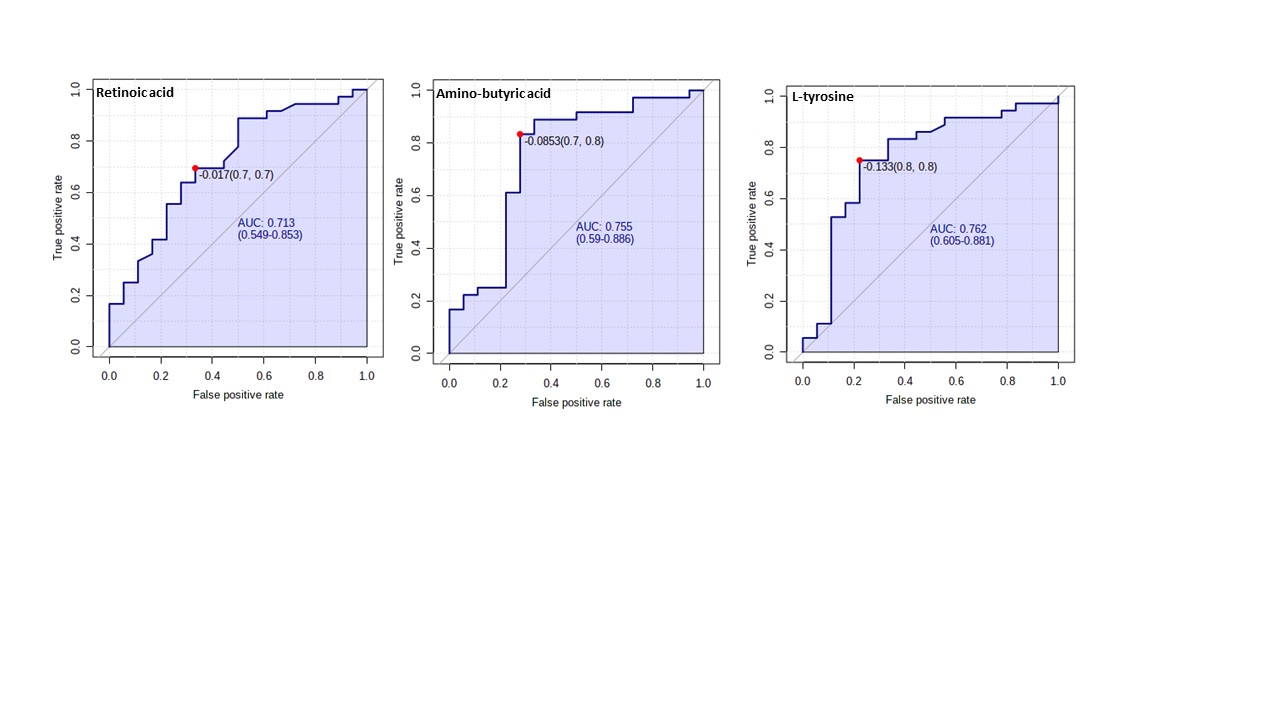
***

***
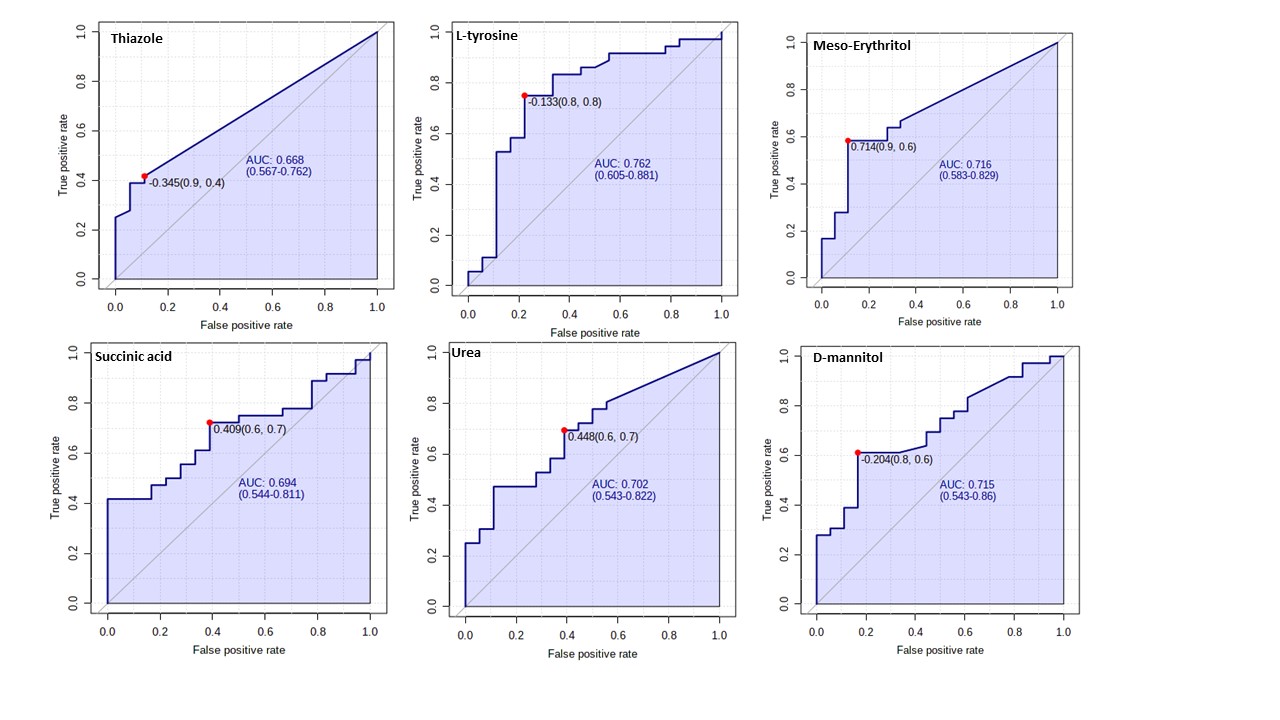
***

**Figure 7**: Verification of the top 15 identified potential biomarkers. ROC analysis to further depict the predictive value of these individual metabolites independently.


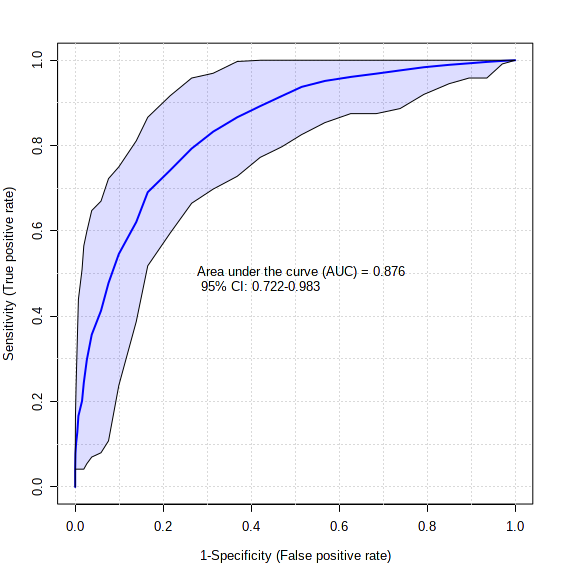


**B**

**A**


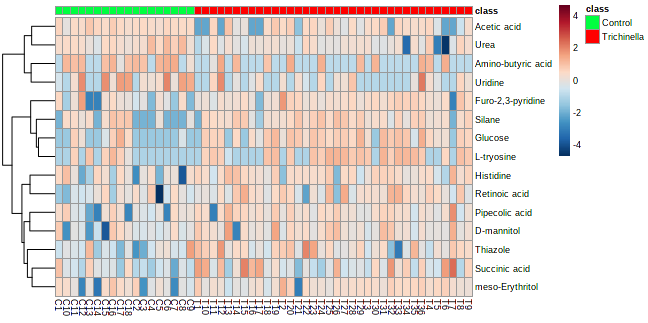


**Figure 8**: A systemic analysis of the key differential biomarkers. A) The receiver-operating curve of the top 15 identified potential biomarkers for distinguishing individuals with *Trichinella zimbabwensis* (Tz) infection. B) Heat map visualization of the key potential biomarkers between the Tz infected and control group.
